# Experimental and Computational Analysis of Newly Synthesized Benzotriazinone Sulfonamides as Alpha-Glucosidase Inhibitors

**DOI:** 10.3390/molecules27206783

**Published:** 2022-10-11

**Authors:** Zunera Khalid, Maha Abdallah Alnuwaiser, Hafiz Adnan Ahmad, Syed Salman Shafqat, Munawar Ali Munawar, Kashif Kamran, Muhammad Mujtaba Abbas, M. A. Kalam, Menna A. Ewida

**Affiliations:** 1 Department of Chemistry, Kinnaird College for Women, Lahore 54000, Pakistan; 2 Department of Chemistry, College of Science, Princess Nourah bint Abdulrahman University, P.O. Box 84428, Riyadh 11671, Saudi Arabia; 3 School of Chemistry, University of the Punjab, Lahore 54590, Pakistan; 4 Department of Chemistry, Division of Science and Technology, University of Education, Lahore 54770, Pakistan; 5 Department of Basic and Applied Chemistry, FAST, University of Central Punjab, Lahore 54000, Pakistan; 6 Department of Physics, University of Agriculture, Faisalabad 38040, Pakistan; 7 Department of Mechanical Engineering, University of Engineering and Technology (New Campus), Lahore 54890, Pakistan; 8 Faculty of Engineering and IT, University of Technology, Sydney 2007, Australia; 9 Department of Pharmaceutical Chemistry, Faculty of Pharmacy, Future University in Egypt, New Cairo 11835, Egypt

**Keywords:** 1,2,3-Benzotriazin-4(3*H*)-one, molecular docking studies, alpha-Glucosidase inhibitors

## Abstract

Diabetes mellitus is a chronic metabolic disorder in which the pancreas secretes insulin but the body cells do not recognize it. As a result, carbohydrate metabolism causes hyperglycemia, which may be fatal for various organs. This disease is increasing day by day and it is prevalent among people of all ages, including young adults and children. Acarbose and miglitol are famous alpha-glucosidase inhibitors but they complicate patients with the problems of flatulence, pain, bloating, diarrhea, and loss of appetite. To overcome these challenges, it is crucial to discover new anti-diabetic drugs with minimal side effects. For this purpose, benzotriazinone sulfonamides were synthesized and their structures were characterized by FT-IR, ^1^H-NMR and ^13^C-NMR spectroscopy. *In vitro* alpha-glucosidase inhibition studies of all synthesized hybrids were conducted using the spectrophotometric method. The synthesized compounds revealed moderate-to-good inhibition activity; in particular, nitro derivatives **12e** and **12f** were found to be the most effective inhibitors against this enzyme, with IC_50_ values of 32.37 ± 0.15 µM and 37.75 ± 0.11 µM. In silico studies, including molecular docking as well as DFT analysis, also strengthened the experimental findings. Both leading compounds **12e** and **12f** showed strong hydrogen bonding interactions within the enzyme cavity. DFT studies also reinforced the strong binding interactions of these derivatives with biological molecules due to their lowest chemical hardness values and lowest orbital energy gap values.

## 1. Introduction

Diabetes mellitus is a metabolic disorder of the endocrine system characterized by hyperglycemia due to impaired insulin tolerance [1,2]. Insulin is the peptide hormone that maintains glucose homeostasis; it binds to cell receptors for the transfer of glucose molecules. This hormone also stimulates fat cells and muscles to obtain glucose from the blood and accelerates the liver for glucose metabolism until the sugar level is lowered to normal [3]. In diabetes mellitus, the pancreas prepares insulin but body cells do not recognize it. As a result, food intake causes hyperglycemia and body cells suffer glucose deficiency [4]. Early symptoms of diabetes are excessive thirst, nausea, shortness of breath, fatigue and frequent urination, but unattended prolonged hyperglycemia can cause other aberrations, including leg amputation, blindness, nerve damage, kidney failure and cardiovascular complications [1,5].

In early times, this disease was known as adult-onset diabetes, but by the end of the twentieth century, it was being widely diagnosed among teens and the pediatric age group. The worldwide prevalence of diabetes among children and young adults is an alarming situation [6]. Diabetes mellitus may be inherent, however, other factors such as smoking, sedentariness, obesity, inactivity and fast food consumption are major causes of this disease [7]. According to the International Diabetes Federation, 536.6 million people were reported to have diabetes in 2021 and this number will increase to 783.2 million by 2045 [8]. Once a patient develops diabetes, that patient suffers from it for the rest of their life [9]. Exercise, weight control and good eating habits can reduce the risk of occurrence. 

In the intestines, alpha-glucosidase is an enzyme that converts food polysaccharides into absorbable glucose, which then enters the blood and increases sugar level [10,11,12]. Therefore, inhibition of alpha-glucosidase is an effective strategy to control hyperglycemia [13]. Commercially, acarbose (Glucobay) and miglitol (Glyset) are famous alpha-glucosidase inhibitors, but daily consumers of these drugs complain of bloating, diarrhea and flatulence, leading to loss of appetite [14,15,16,17,18]. To overcome these side effects, it is crucial to explore new alpha-glucosidase inhibitors with better inhibition potency and minimal side effects.

**Figure 1:**
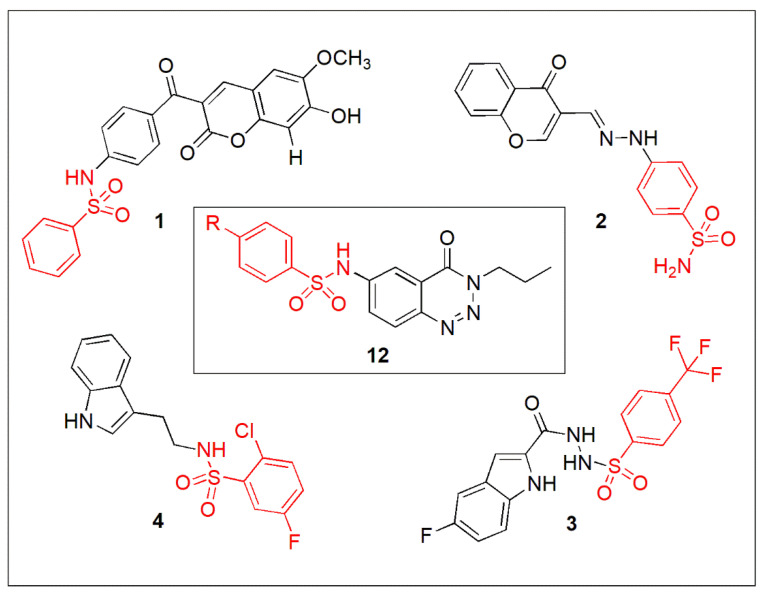
Reported structures of heterocyclic sulfonamides as alpha-glucosidase inhibitors.

Sulfonamide is a famous pharmacophore responsible for several biological activities; it is a part of many clinical drugs [19,20]. Different heterocyclic groups with sulfonamide functionality have been studied as alpha-glucosidase inhibitors. Coumarin sulfonamide **1** [21], chromone sulfonyl hydrazone **2** [21], indole sulfonyl hydrazide **3** [22] and indole sulfonamide **4** [23] have been reported to be strong alpha glucosidase inhibitors (Figure 1). 1,2,3-Benzotriazin-4(3*H*)-one is known for its biologically active nucleus, and its derivatives have been reported as anti-inflammatory [24], anti-depressant [25], anticancer [26], anti-diarrheal [27], HIV inhibitor [28] and as anesthetics [29]. Some of its derivatives had been studied as enzyme inhibitors of 4-hydroxyphenylpyruvate dioxygenase [30], matrix metalloprotease [31], alpha-glucosidase [15], chorismate mutase [32], HepG2 [33] and acetylcholinesterase [34]. 

**Figure 2:**
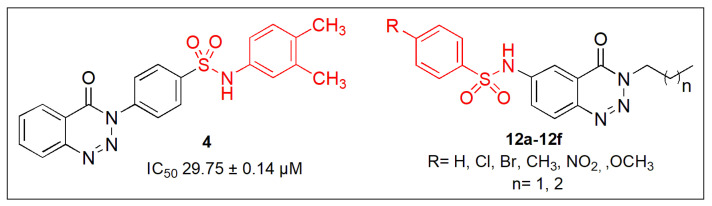
Design of new 1,2,3-benzotriazin-4(3*H*)-one sulfonamides **12a–12f**.

In our efforts to develop an anti-diabetic drug with an enhanced therapeutic effect, we reported a series of benzotriazinone sulfonamide derivatives as potent alpha-glucosidase inhibitors, and one of them, compound **4** (Figure 2), exhibited good results, with an IC_50_ value of 29.75 ± 0.14 μM [15]. In order to further enhance the potency of compound **4**, our group designed another series of benzotriazinone sulfonamide derivatives (**12a–f**). This design mainly involved the replacement of the aryl group of compound **4** by alkyl moiety at the third position, as well as the introduction of sulfonamide moiety at the sixth position of benzotriazinone (Figure 2). Novel hybrids of 4-phenyl-*N*-(4-oxo-3-alkyl-3,4-dihydrobenzo[1,2,3]triazin-6-yl)benzenesulfonamide (**12a–f**) were prepared and their in vitro inhibition potential was studied in comparison to the commercial drug acarbose. Moreover, molecular docking analysis was employed to explain *in vitro* studies and to explore the binding mode of interactions of all hybrids against the binding sites of target alpha glucosidase. Furthermore, the stability and reactivity of the screened compounds were calculated by DFT studies to explore their hybrid interactions with other molecules.

## 2. Results and Discussion

### 2.1. Synthetic Chemistry

**Scheme 1:**
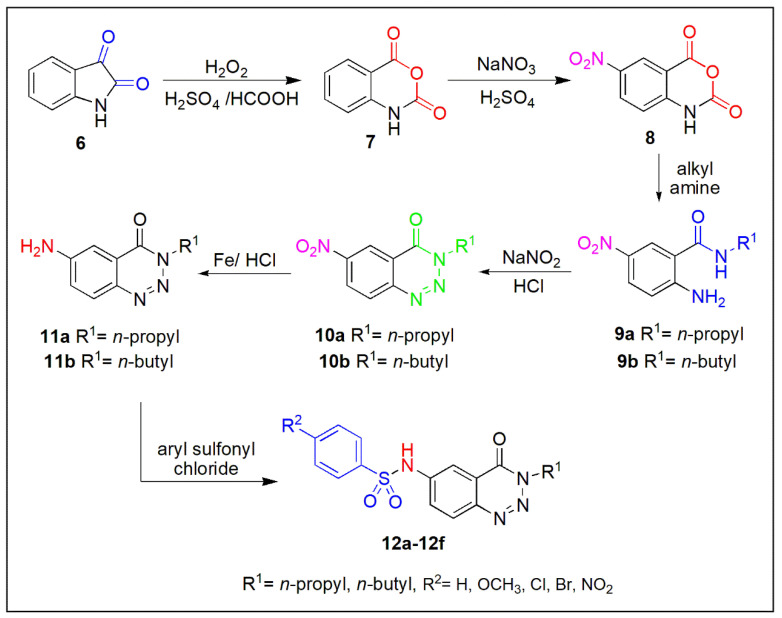
Synthetic route for the synthesis of 1,2,3-benzotriazin-4(3*H*)-one based sulfonamides.

1,2,3-Benzotriazin-4(3*H*)-one-based sulfonamides (**12a–12f**) were synthesized by using isatin (**6**) as an inexpensive starting material; it was initially oxidized to isatoic anhydride (**7**) and then nitrated in a sodium nitrate/sulfuric acid mixture to prepare 6-nitroisatoic anhydride (**8**) followed by reaction with *n*-propyl and *n*-butylamine to prepare their corresponding anthranilamides (**9a**, **9b**, Scheme 1). Further cyclization was conducted in a nitrous acid mixture to yield **10a** and **10b** followed by reduction with Fe/HCl mixture. In the final step, 6-amino-*N*-propyl-1,2,3-benzotriazin-4(3*H*)-one (**11a**) and 6-amino-*N*-butyl-1,2,3-benzotriazin-4(3*H*)-one (**11b**) were reacted with different arylsulfonyl chlorides to accomplish various sulfonamide hybrids **12a–12f** (Scheme 1).

### 2.2. Spectroscopic Characterization

In multistep synthesis, the starting compounds **7**, **8**, **9a, 9b**, **10a** and **10b** were prepared and characterized by FTIR spectral analysis, and observations were consistent with the values of similar compounds found in the literature [35,36]. The FTIR spectrum of isatoic anhydride **7** displayed strong absorption bands at 1722, 1765 cm^−1^ (C=O) and 1021 cm^−1^ (C-O-C), indicating the presence of anhydride moiety [35]. Regarding nitration, the presence of the NO_2_ group in compound **8** was identified by FTIR band at 1355 cm^−1^ [36]. The anhydride group of compound **8** was reacted with alkyl amines, and as a result, *N*-alkyl anthranilamides **9a** and **9b** were formed as products, which were confirmed by the appearance of amino group IR bands at 3293–3318 cm^−1^ [37]. Further cyclization of anthranilamides was confirmed by the disappearance of NH_2_ bands in both nitro benzotriazinones **10a** and **10b** [38]. Reduction of the NO_2_ group to NH_2_ was confirmed by the appearance of an IR band at 3430 cm^−1^ [39]. In sulfonamides (**12a–12f**), symmetrical stretching vibrational bands of S=O and NH were observed at 1157–1187 and 3185–3221 cm^−1^ [15]. Amino products **11a** and **11b** and all final products (**12a–12f**) were further characterized by ^1^H-NMR and ^13^C-NMR spectroscopic techniques. The amino group of **11a** and **11b** appeared at 6.56 ppm, while after conversion into the sulfonamide group, the NH group was noted to be in the range of 11.35–11.47 ppm. ^1^H-NMR analysis of **11a**, **11b** and **12a–12f** revealed the propyl protons as triplet, multiplet and triplet in the range of 0.84–4.82 ppm and the aromatic protons of phenyl rings were observed in the range of 7.02–8.12 ppm. Further ^13^C-NMR spectral results confirmed the structure of all synthesized compounds. 

### 2.3. Alpha-Glucosidase Inhibition Studies

Acarbose is a commercially available antidiabetic drug used to treat diabetes mellitus. It actively works in the intestinal tract by inhibiting alpha-glucosidase, which is responsible for the conversion of complex carbohydrates to absorbable glucose. Acarbose has various side effects and chemists are working to discover a new effective anti-diabetic drug. Therefore, an alpha-glucosidase inhibition assay of newly synthesized amino benzotriazinones **11a–11b** and their corresponding sulfonamides **12a–12f** was carried out by spectrophotometric method. Comparative results of all compounds (**12a–12f)** with reference to standard drug acarbose are given in Table 1. The results proved to have fair to good inhibition activity. From Table 1, it is evident that amino benzotriazinones **11a** and **11b** exhibited less inhibition in contrast to their sulfonamides, which indicated the effective interactions of sulfonyl group within the enzyme cavity. Nitro derivative **12e** revealed the most promising activity, with an IC_50_ value of 32.37 ± 0.15 μM as compared to the standard drug acarbose, with an IC_50_ value of 37.38 ± 0.12 μM. Another nitro compound (**12f**) with the butyl group was found to be an equivalent inhibitor of acarbose, with an IC_50_ value of 37.75 ± 0.11 μM.

The difference in the inhibition potentials of **12e** and **12f** illustrated that the small chain of the propyl group is more appropriate to be fitted with an alpha-glucosidase structure. Electron density on oxygen atoms of the nitro group enabled it to have a strong inhibitory potential. Different groups at the fourth position of the phenyl ring revealed different inhibition strengths. Compound **12a** indicated a lesser enzyme inhibition potency as compared to other compounds having Cl, Br, NO_2_ and OCH_3_ groups. The lone pair containing substituents on the phenyl ring supported inhibition activity due to their electronic interactions with enzyme (active) sites. The larger size of the bromine atom reduced the inhibition activity of compound **12d** (IC_50_ value 55.21 ± 0.12 μM) as compared to the chloro-containing compound **12c** (IC_50_ value 53.27 ± 0.13 μM). The methoxy derivative **12b** was found to be more effective inhibitor as compared to **12a** but less active than **12e** and **12f**.

### 2.4. Molecular Docking

The probable binding interactions of the tested compounds within the enzyme cavity were explored by molecular docking studies. Autodoc Vina and its tools were utilized to elaborate all types of interactions. The receptor file of alpha-glucosidase was prepared by homology modeling, as reported in our previous work [15]. Chemdraw office pack was utilized to prepare and optimize the ligand’s structure by using the MM2 force field.

**Figure 3:**
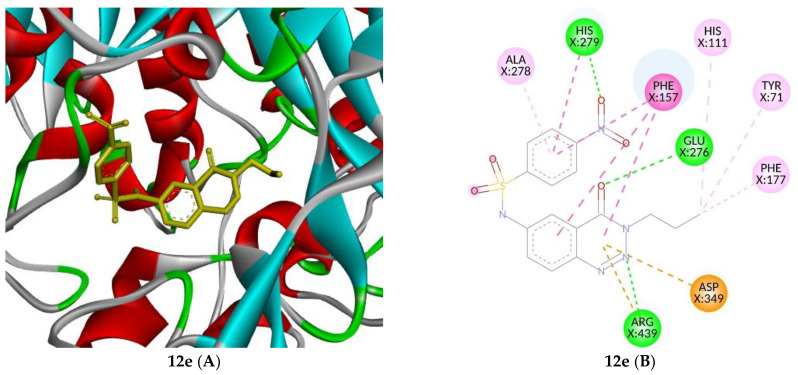
2D (**B**) and 3D (**A**) structures illustrating docking results, ligand **12e** within alpha-glucosidase develops the interactions shown by colored lines; hydrogen bonding (green), pi-cationic/pi anionic interaction (brown), pi-pi interaction (pink) and pi-alkyl interaction (purple).

**Figure 4:**
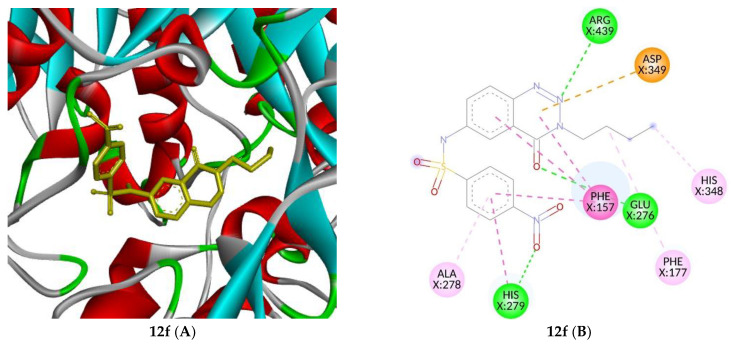
2D (**B**) and 3D (**A**) structures illustrating docking results, ligand **12f** within alpha-glucosidase develops interactions shown by colored lines; hydrogen bonding (green), pi-cationic/pi anionic interaction (brown), pi-pi interaction (pink) and pi-alkyl interaction (purple).

Both **12e** and **12f** formed stable ligand enzyme complexes and displayed the highest binding energy, −9.5 and −9.3 kcal.mol^−1^, respectively. Binding pose of the docked conformation with highest binding affinity of both ligands **12e** and **12f** are presented in Figure 3 and Figure 4, respectively. From these figures, it is evident that both ligands are fit inside the enzyme pockets due to different kinds of binding interactions, including electrostatic, hydrogen bonding as well as hydrophobic interactions. In these two cases, the highest binding may be associated with the development of hydrogen bonding between the carbonyl group of ligands and the carboxylic group of GLU276. The nitro group of **12e** and **12f** also established hydrogen bonding with the NH group of HIS279. Similarly, one conventional hydrogen bond can be seen between nitrogen of the benzotriazinone ring and the NH group of the ARG439 amino acid (Figure 3 and Figure 4).

Similarly, the benzotriazinone ring of both ligands (**12e** and **12f)** depicted pi-anionic electrostatic interaction with the carboxylic acid group of ASP349. However, an additional pi-cationic interaction between the NH group of ARG349 and the benzotriazinone ring was found in the case of compound **12e** (Figure 3 and Figure 4).

All rings, along with alkyl groups of both ligands **12e** and **12f**, established various kinds of hydrophobic interactions with the residues of different amino acids (Figure 3 and Figure 4). Significantly, pi-pi T-shaped interactions appeared between pi-electron densities of both ligands and aromatic side chains of PHE157 and HIS279. Similarly, pi-alkyl interactions between the methyl group of ALA278 and the ring density of the benzene ring attached with the nitro group can be observed in the case of both ligands. All mentioned forces made strong ligand enzyme complexes that enabled these compounds (**12e** and **12f**) to act as effective alpha-glucosidase inhibitors. Molecular docking results explored that both **12e** and **12f** docked compounds have almost similar kinds of interactions within the active pockets of enzyme (Figure 3 and Figure 4). However, the relatively lesser activity of compound **12f** as compared to **12e** can be explained in terms of increasing hydrophobicity of the ligand due to the increasing chain of the alkyl group in compound **12f** that makes the ligand–enzyme complex relatively less stable, as observed in the binding energies.

### 2.5. Computational Study

#### 2.5.1. Frontier Molecular Orbital Analysis

Quantum chemistry calculations regarding the highest occupied molecular orbital (HOMO) and lowest unoccupied molecular orbital (LUMO) are collectively known as frontier molecular orbital analyses (FMO). FMO analyses are helpful in drug design and in determining reactivity. HOMO is the highest energy orbital of molecule with electrons while LUMO is the lowest energy orbital of molecule with no electrons [40]. Energy difference between HOMO and LUMO is the main factor for compound reactivity. All synthesized compounds **12a–12f** were studied by frontier molecular orbital analysis and results supported that all synthesized molecules have high chemical reactivity [41,42].

Energy of HOMO (E_HOMO_), LUMO (E_LUMO_) and energy gaps (E_gap =_ HOMO–LUMO) between them were calculated by B3LYP/6-311+G* level of DFT method (Table 2). Large energy gap value illustrates that compound is less reactive while small energy gap indicates the less kinetic stability and high chemical reactivity. Among all compounds, **12e** and **12f** were found to have lowest energy gap values revealing that these compounds are more reactive as compared to others. Furthermore, the HOMO-LUMO energy analysis of all compounds provided valuable information about their global reactivity descriptors like E_HOMO_, E_LUMO_, electrophilicity, chemical potential, chemical softness and various other electronic parameters which are tabulated in Table 2 [40,43].

The electrophilicity values (**ω**) of all compounds explained their bonding, stability, structure and reactivity [40,43]. Compounds **12e** and **12f** presented the highest values of electrophilicity, 7.25 and 7.26 eV, which is evidence of their greater interactions with biomolecules. Electrophilicity index with molecular docking studies supported the in vitro alpha-glucosidase inhibition results where **12e** and **12f** exhibited good inhibitory activity.

**Figure 5:**
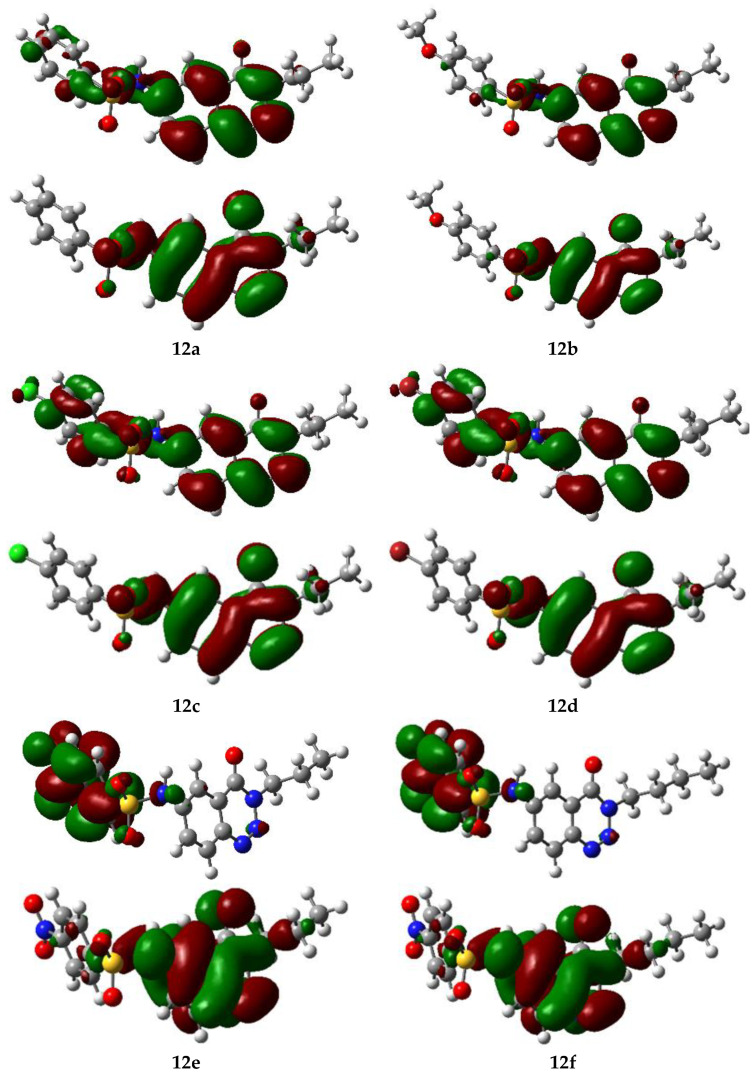
Pictorial illustration of charge density distribution in molecular orbitals of compounds **12a–12f**. HOMO is shown at the bottom and LUMO is displayed at the top.

For the synthesized series **12a–12f,** delocalization of both HOMO and LUMO was identified by charge density, as demonstrated in Figure 5. It was observed that HOMO is located on the site of the benzotriazinone ring, and sulfonamide moiety and LUMO delocalization is different in different molecules. The compound with no substitution, **12a,** as well as the compound with an electron donating group, **12b**, exhibited their LUMO delocalizations confined on the benzotriazinone ring. While in compounds **12c** and **12d**, the electron density of LUMO was delocalized on their phenyl ring and sulfonamide moiety. However, **12a**, **12b** and **12d** have LUMO delocalization on the surface of the benzotriazinone ring and sulfonamide group (Figure 5). The compounds **12c, 12d, 12e** and **12f** have electron withdrawing groups (Cl, Br and NO_2_), restraining LUMO electron density only on the phenyl ring.

#### 2.5.2. Molecular Electrostatic Potential

**Figure 6:**
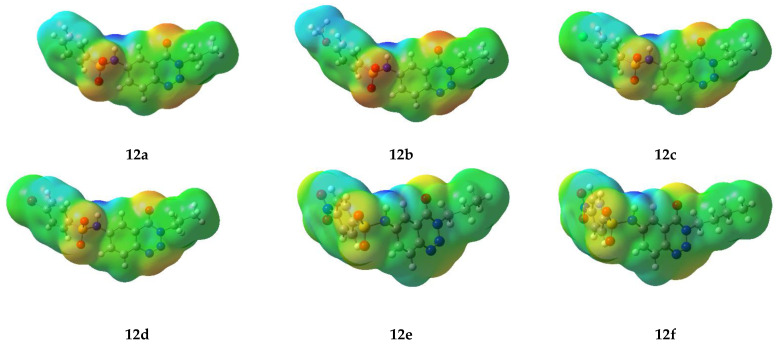
Electrostatic potential on the surfaces of 1,2,3-benzotriazin-4(3*H*)-one sulfonamides.

At the molecular level, MEP is an effective method to describe the electron density distribution map inside molecules and can illustrate an idea about the reactive sites of molecular structure. This method provides images according to the relative polarity present in a molecule, which indicate the electrophilic portion (negative site) and nucleophilic portion (positive side) of the compound. MEP studies were carried out using a B3LYP/6-311+G* method and the results are shown in Figure 6. Surface maps displayed pictures with different colors, indicating different electrostatic potential values. The positive sites are shown by the blue colour, while the yellow colour depicts slightly positive areas, and neutral electrostatic potential regions are represented by green. The positive sites indicated by the blue colour of all molecules tend to repel protons. Further, this study helped to identify reactive sites of chemical systems and indicated the sites where ligand can bind to enzyme. MEP analysis of all compounds depicted the existence of strong positive potential on hydrogen atoms of sulfonamide functionality and partial positive charge on C=O, N=N and SO_2_ groups. The existence of strong and partial positive charges on functional groups of synthesized series have a tendency to develop appropriate interactions within the enzyme cavity.

## 3. Experiment Work

### 3.1. General

All chemicals were purchased from international supplier, Sigma-Aldrich (USA and Germany), Fluka (Switzerland). Merck TLC plates (Germany) were used to monitor the progress of reactions under short and long wavelengths (model UVGL-25 minor light multiband UV-254/366). VWR formic acid (USA) and Merck glacial acetic acid (Germany) were used in the synthesis of isatoic anhydride. Melting points were recorded on the Fisher–Johns apparatus (University of the Punjab, Lahore), calibrated by benzoic acid. The FTIR spectra were taken on Agilent 630 FTIR (University of the Punjab, Lahore). In NMR (H.E.J Research institute of chemistry, University of Karachi), TMS was used as internal standard and all spectra were recorded in CDCl_3_ or DMSO-d_6_ on Brucker (300, 400 and 500 MHz).

#### 3.1.1. Synthesis of Isatoic Anhydride (7)

Isatin (**6**, 14.7 g, 0.1 mmol) and formic acid (80 mL) were taken in an Erlenmeyer flask. Hydrogen peroxide (30%, 20 mL) was added slowly with slight cooling, and the reaction mixture was stirred for 1h at room temperature. Precipitates of product appeared in the flask and were filtered and recrystallized from methanol. Light yellow compound; yield: (13.85 g, 85%); m.p. 230–232 ℃ (Lit. m.p. with decomposition: 235 °C [36]. FT-IR (v-cm^−1^): 1021 (C-O-C), 1722 (C=O), 1765 (C=O), 3236 (N-H).

#### 3.1.2. 6-Nitroisatoic Anhydride (8)

Isatoic anhydride (**7**, 3.42 g, 21 mmol) and chilled concentrated sulfuric acid (98%, 18.4 M, 75 mL) were taken in a round bottom flask (250 mL). Sodium nitrate (2.22 g, 26.25 mmol) was added in small portions and ice-bath was used to maintain the temperature below 5 °C. Then, the reaction mixture was stirred for 15 min and poured off into ice-cold water (500 mL). Precipitates formed on slight stirring were filtered, dried and recrystallized from ethanol. Yellow powder; yield: (2.45 g, 62%); m.p. 219–221 °C (Lit. m.p. 220 °C) [44]. FT-IR (v-cm^−1^): 1355 (NO_2_), 1578 (NO_2_), 1680 (C=O), 1738 (C=O), 3198 (N-H).

#### 3.1.3. 2-Amino-5-nitro-N-alkylbenzamides (9a–9b)

6-nitroisatoic anhydride (**8**, 4 g, 19.2 mmol) and alkylamine (19.2 mmol) were mixed in 1,4-dioxan and heated under reflux. After 3h, the solvent was evaporated under reduced pressure and the residue was recrystallized from methanol and further dried at 70 °C.

*2-Amino-5-nitro-N-propylbenzamide* (**9a**). Propylamine (1.58 mL, 19.2 mmol) and 6-nitroisatoic anhydride (**8**, 4 g, 19.2 mmol) were used to prepare mustard powder; yield: (2.80 g, 60%); m.p. 195–197 °C. FT-IR (v-cm^−1^): 1334 (NO_2_), 1522 (NO_2_), 1608 (C=O), 3293 (NH_2_).

*2-Amino-5-nitro-N-butylbenzamide* (**9b**). Butylamine (1.87 mL, 19.2 mmol) and 6-nitro isatoic anhydride (**8**, 4 g, 19.2 mmol) were used to synthesize mustard powder; yield: (2.75 g, 58%); m.p. 197–199 °C. FT-IR(v-cm^−1^): 1333 (NO_2_), 1536 (NO_2_), 1650 (C=O), 3318 (NH_2_).

#### 3.1.4. General Procedure for the Synthesis of 6-Nitro-3-alkylbenzo[1,2,3]triazin-4(3*H*)-ones (10a–10b)

In a conical flask, compound **9a/9b** (3 g, 12.8 mmol) was taken in chilled water (20 mL), and HCl solution (30%, 5 mL) was added into it. The reaction mixture was placed in an ice bath to maintain the temperature at 0–5 °C. Sodium nitrite (16.6 mmol) was dissolved in distilled water (10 mL) and this solution was slowly added to the reaction mixture; the temperature was maintained below 5 °C. Afterwards, the reaction was stirred for two hours and ice-cold water was added to obtain precipitates of product. The product was washed twice with chilled water and recrystallized from methanol followed by drying at 70 °C.

*6-Nitro-3-propylbenzo[1,2,3]triazin-4(3H)-one***(10a)**. Compound **9a** (3 g, 12.8 mmol), HCl solution (30%, 5 mL) and sodium nitrite (1.16 g, 16.6 mmol) were used to obtain light yellow powder; yield: (2.80 g, 92%); m.p. 215–217 °C. FT-IR (v-cm^−1^): 1344 (NO_2_), 1530 (NO_2_), 1687 (C=O), 2968 (CH_2_).

*6-Nitro-3-butylbenzo[1,2,3]triazin-4(3H)-one***(10b)**. Compound **9b** (3 g, 12.8 mmol), HCl solution (30%, 5 mL) and sodium nitrite (1.13 g, 16.6 mmol) were used to prepare white powder; yield: (2.75 g, 91%); m.p. 217–218 °C. FT-IR (v-cm^−1^): 1341 (NO_2_), 1535 (NO_2_), 1591 (C=O), 2962 (CH_2_).

#### 3.1.5. General Procedure for the Synthesis of 6-Amino-3-alkylbenzo[1,2,3]triazin-4(3*H*)-one (11a–11b)

Compound **10a–10b** (0.967 mmol), iron powder (2.42 g, 10 mmol) and HCl (30%, 5 mL) solution were taken in water (10 mL). Reaction mixture was stirred for two hours at 60 °C. Afterwards, the reaction mixture was filtered and the filtrate was cooled until crystals of product appeared. It was then recrystallized with chloroform.

*6-Amino-3-propylbenzo[1,2,3]triazin-4(3H)-one***(11a)**. Compound **10a** (0.15 g, 0.967 mmol), iron powder (2.42 g, 10 mmol) and HCl (30%, 5 mL) solution were used to obtain transparent crystals; yield: (0.11 g, 80%); m.p. 185–186 ℃. FT-IR (v-cm^−1^): 1255 (C-N), 1638 (C=O), 2957 (CH_2_), 3435 (NH_2_); ^1^H-NMR: (CDCl_3,_ 500 MHz): δ_H_ 0.891 (t, *J* = 7.5 Hz, 3H, CH_3_), 1.73–1.81 (m, 2H, CH_2_), 4.22 (t, *J* = 7.2 Hz, 2H, CH_2_), 6.56 (s, 2H, NH_2_), 7.16 (dd, *J*= 10.0, 2.5 Hz, 1H, ArH), 7.19 (d, *J* = 3.0 Hz, 1H, ArH), 7.81 (d, *J* = 8.5 Hz, 1H, ArH). ^13^C-NMR: (DMSO-d_6_, 126 MHz): δ_C_ 11.5 (C, NCH_2_CH_2_*C*H_3_), 22.3 (C, NCH_2_*C*H_2_CH_3_), 50.6 (C, N*C*H_2_CH_2_CH_3_), 103.4 (CH, C-5), 121.9 (CH, C-7), 122.3 (C, C-4a), 130.4 (CH, C-8), 136.2 (C, C-8a), 153.6 (C, C-6), 155.6 (CO, C-4).

*6-Amino-3-butylbenzo[1,2,3]triazin-4(3H)-one* **(11b)**. Compound **10b** (0.24 g, 0.967 mmol), iron powder (2.42 g, 10 mmol) and HCl (30%, 5 mL) solution were used to prepare transparent crystals; yield: (0.18 g, 72%); m.p. 191–192 ℃. FT-IR (v-cm^−1^): 1256 (C-N), 1693 (C=O), 2916 (CH_2_), 3435 (NH_2_); ^1^H-NMR: (CDCl_3_, 500 MHz): δ_H_ 0.85 (t, *J* = 7.3 Hz, 3H, CH_3_), 1.25–1.28 (m, 2H, CH_2_), 1.66–1.71 (m, 2H, CH_2_), 4.22 (t, *J* = 7.2 Hz, 2H, CH_2_), 6.55 (s, 2H, NH_2_), 7.12 (d, *J* = 2.4 Hz, 1H, ArH), 7.15 (dd, *J* = 8.8, 2.5 Hz, 1H, ArH), 7.78 (d, *J* = 8.8 Hz, 1H, Ar-*H*). ^13^C-NMR: (DMSO-d_6_, 126 MHz): δ_C_ 14.0 (C, NCH_2_CH_2_CH_2_*C*H_3_), 19.8 (C, NCH_2_CH_2_*C*H_2_CH_3_), 31.0 (C, NCH_2_*C*H_2_CH_2_CH_3_), 48.7 (C, N*C*H_2_CH_2_CH_2_CH_3_), 103.4 (CH, C-5), 121.9 (CH, C-7), 122.3 (C, C-4a), 130.4 (CH, C-8), 136.2 (C, C-8a), 153.6 (C, C-6), 155.6 (CO, C-4).

#### 3.1.6. General Procedure for the Synthesis of N-(4-Oxo-3-propyl/butyl-3,4-dihydrobenzo[1,2,3]triazin-6-yl)benzenesulfonamides (12a-12f)

Compound **11a/11b** (0.1 g, 0.49 mmol), benzene sulfonyl chloride (0.49 mmol) and pyridine (10 mL) were taken in a 50 mL round bottom flask and refluxed for 5 h. Then, the pyridine was evaporated under reduced pressure and the mixture was cooled to room temperature. A few drops of dilute HCl were added to ice-cold water (100 mL) until the pH reached 3, and the temperature was maintained at 5 °C. Further, the reaction mixture was poured off into acidified water. Precipitates appearing in the solution were filtered, dried and recrystallized from methanol. The physiochemical properties of all synthesized compounds are given in Table 3.

*N-(4-Oxo-3-propyl-3,4-dihydrobenzo[1,2,3]triazin-6-yl)benzenesulfonamide* **(12a)**. Benzenesulfonyl chloride (0.15 g, 0.49 mmol) and compound **11a** (0.1 g, 0.49 mmol) were used to prepare brown powder; yield: (0.18 g, 82%); m.p. 171–173 ℃. FT-IR (v-cm^−1^): 1167 (S=O), 1331 (S=O), 1659 (C=O), 2964 (CH_2_), 3185 (N-H); ^1^H-NMR: (CDCl_3_, 300 MHz): δ_H_ 1.01 (t, *J* = 7.5 Hz, 3H, CH_3_), 1.90–1.97 (m, 2H, CH_2_), 4.82 (t, *J* = 7.2 Hz, 2H, CH_2_,), 7.44 (t, *J* = 7.3 Hz, 2H, ArH), 7.52 (t, *J* = 7.2 Hz, 1H, ArH), 7.90 (d, *J* = 7.5 Hz, 2H, ArH), 7.93–8.12 (m, 3H, ArH), 8.59 (s, 1H, NH). ^13^C-NMR: (DMSO-d_6_, 126 MHz): δ_C_ 11.5 (C, NCH_2_CH_2_*C*H_3_), 22.1 (C, NCH_2_*C*H_2_CH_3_), 51.2 (C, N*C*H_2_CH_2_CH_3_), 111.3 (CH, C-5), 120.9 (CH, C-7), 126.1 (C, C-4a), 127.2 (2CH, C-2′, C6′), 130.2 (2CH, C-3′, C-5′), 130.5 (CH, C-8), 134.1 (CH, C-4′), 139.4 (C, C-6), 140.4 (C, C-8a), 142.1 (C, C-1′), 155.0 (CO, C-4).

*4-Methoxy-N-(4-oxo-3-propyl-3,4-dihydrobenzo[1,2,3]triazin-6-yl)benzenesulfonmide***(12b)**. 4-Methoxybenzenesulfonyl chloride (0.11 g, 0.49 mmol) and compound **11a** (0.1 g, 0.49 mmol) were used to synthesize brown powder; yield: (0.13 g, 75%); m.p. 175–177 ℃. FT-IR (v-cm^−1^): 1158 (S=O), 1333 (S=O), 1664 (C=O), 2964 (CH_2_), 3222 (N-H); ^1^H-NMR: (DMSO-d_6,_ 500 MHz): δ_H_ 0.84 (t, *J* = 7.5 Hz, 3H, CH_3_), 1.71–1.76 (m, 2H, CH_2_), 3.73 (s, 3H, OCH_3_), 4.22 (t, *J* = 7.3 Hz, 2H, CH_2_), 7.05 (d, *J* = 9.0 Hz, 2H, ArH), 7.69–7.81 (m, 4H, ArH), 8.06 (d, *J* = 2.1 Hz, 1H, ArH), 11.22 (s, 1H, NH). ^13^C-NMR: (DMSO-d_6_, 126 MHz): δ_C_ 11.5 (C, NCH_2_CH_2_*C*H_3_), 22.1 (C, NCH_2_*C*H_2_CH_3_), 51.2 (C, N*C*H_2_CH_2_CH_3_), 56.2 (C, O*C*H_3_), 111.0 (CH, C-5), 115.3 (2CH, C-3′, C-5′), 120.9 (CH, C-7), 125.9 (C, C-4a), 129.5 (2CH, C-2′, C-6), 130.4 (CH, C-8), 130.9 (C, C-1′), 140.3 (C, C-6), 142.4 (C, C-8a), 155.0 (CO, C-4), 163.4 (C, C-4′).

*4-Chloro-N-(4-oxo-3-propyl-3,4-dihydrobenzo[1,2,3]triazin-6-yl)benzenesulfonamide***(12c)**. 4-Chlorobenzenesulfonyl chloride (0.1 g, 0.49 mmol) and compound **11a** (0.1 g, 0.49 mmol) were used to prepare brown powder; yield: (0.13 g, 68%); m.p. 169–171 °C. FT-IR (v-cm^−1^): 1161 (S=O), 1333 (S=O), 1656 (C=O), 2961 (CH_2_), 3193 (NH); ^1^H-NMR: (DMSO-d_6,_ 500 MHz): δ_H_ 0.84 (t, *J* = 7.2 Hz, 3H, CH_3_), 1.74–1.77 (m, 2H, CH_2_), 4.22 (t, *J* = 7.1 Hz, 2H, CH_2_), 7.70–7.78 (m, 5H, ArH), 7.81 (d, *J* = 1.5 Hz, 1H, ArH), 8.08 (d, *J* = 8.5 Hz, 1H, ArH), 11.40 (s, 1H, NH). ^13^C-NMR: (DMSO-d_6_, 126 MHz): δ_C_ 11.5 (C, NCH_2_CH_2_*C*H_3_), 22.1 (C, NCH_2_*C*H_2_CH_3_), 51.2 (C, N*C*H_2_CH_2_CH_3_), 111.7 (CH, C-5), 121.0 (CH, C-7), 126.3 (C, C-4a), 128.1 (CH, C-8), 129.2 (2CH, C-2′, C-6′), 130.5 (C, C-6), 133.3 (2CH, C-3′, C-5′), 138.7 (C, C-4′), 140.6 (C, C-1′), 141.8 (C, C-8a), 155.0 (CO, C-4).

*4-Bromo-N-(4-oxo-3-propyl-3,4-dihydrobenzo[1,2,3]triazin-6-yl)benzenesulfonamide***(12d)**. 4-Bromobenzenesulfonyl chloride (0.13 g, 0.49 mmol) and compound **11a** (0.1 g, 0.49 mmol) were used to obtain brown powder; yield: (0.14 g, 78%); m.p. 177–179 ℃. FT-IR (v-cm^−1^): 1164 (S=O), 1332 (S=O), 1657 (C=O), 2972 (CH_2_), 3181 (N-H); ^1^H-NMR: (DMSO-d_6,_ 500 MHz) δ_H_: 0.85 (t, *J* = 7.4 Hz, 3H, CH_2_), 1.70–1.78 (m, 2H, CH_2_), 4.23 (t, *J* = 7.4 Hz, 2H, CH_2_), 7.62 (d, *J* = 8.0 Hz, 2H, ArH), 7.70 (dd, *J* = 8.8 Hz, 2.5 Hz, 1H, ArH), 7.81–7.83 (m, 3H, ArH), 8.08 (d, *J* = 8.8 Hz, 1H, ArH), 11.38 (s, 1H, NH). ^13^C-NMR: (CDCl_3_, 100 MHz): δ_C_ 11.7 (C, NCH_2_CH_2_*C*H_3_), 22.2 (C, NCH_2_*C*H_2_CH_3_), 51.0 (C, N*C*H_2_CH_2_CH_3_), 111.7 (CH, C-5), 120.0 (CH, C-7), 126.3 (C, C-4a), 128.1 (CH, C-8), 129.2 (2CH, C-2′, C-6′), 130.5 (2CH, C-3′, C-5′), 133.3 (C, C-6), 138.7 (C, C-4′), 140.6 (C, C-1′), 141.8 (C, C-8a), 158.0 (CO, C-4).

*4-Nitro-N-(4-oxo-3-propyl-3,4-dihydrobenzo[1,2,3]triazin-6-yl)benzenesulfonamide***(12e)**. 4-Nitrobenzenesulfonyl chloride (0.11 g, 0.49 mmol) and compound **11a** (0.1 g, 0.49 mmol) were used to obtain brown powder; yield: (0.14 g, 71%); m.p. 167–169 °C. FT-IR (v-cm^−1^): 1161 (S=O), 1357 (S=O), 1696 (C=O), 2932 (CH_2_), 3210 (NH); ^1^H-NMR: (DMSO-d_6,_ 500 MHz) δ_H_: 0.85 (t, *J* = 7.3 Hz, 3H, CH_3_), 1.72–1.76 (m, 2H, CH_2_), 4.23 (t, *J* = 7.0 Hz, 2H, CH_2_), 7.70–7.78 (m, 5H, ArH), 7.81 (d, *J* = 1.5 Hz, 1H, ArH), 8.08 (d, *J* = 8.8 Hz, 1H, ArH), 11.36 (s, 1H, NH). ^13^C-NMR: (DMSO-d_6_, 126 MHz): δ_C_ 11.5 (C, NCH_2_CH_2_*C*H_3_), 22.1 (C, NCH_2_*C*H_2_CH_3_), 51.2 (C, N*C*H_2_CH_2_CH_3_), 111.3 (CH, C-5), 120.9 (CH, C-7), 126.1 (C, C-4a), 127.2 (2CH, C-3′, C-5′), 130.2 (2CH, C-2′, C-6′), 130.5 (CH, C-8), 134.1 (C, C-6), 139.4 (C, C-8a), 140.4 (C, C-1′), 142.1 (C, C-4′), 155.0 (CO, C-4).

*4-Nitro-N-(4-oxo-3-butyl-3,4-dihydrobenzo[1,2,3]triazin-6 yl)benzenesulfonamide***(12f)**. 4-Nitrobenzenesulfonyl chloride (0.12 g, 0.49 mmol) and compound **11b** (0.1 g, 0.49 mmol) were used to obtain brown powder; yield: (0.06 g, 62%); m.p. 179–181 °C. FT-IR (v-cm^−1^): 1189 (S=O), 1380 (S=O), 1597 (C=O), 2919 (CH_2_), 3221 (NH); ^1^H-NMR: (DMSO-d_6,_ 500 MHz) δ_H_: 0.84 (t, *J* = 7.4 Hz, 3H, CH_3_), 1.66–1.79 (m, 2H, CH_2_), 2.20–2.24 (m, 4H, CH_2_), 4.23–4.27 (m, 2H, CH_2_), 7.34 (m, 2H, ArH), 7.71–7.81 (m, 4H, ArH), 8.04 (s, 1H, ArH), 11.32 (s, 1H, NH). ^13^C-NMR: (CDCl_3_, 100 MHz): δ_C_ 11.5 (C, NCH_2_CH_2_CH_2_*C*H_3_), 21.5 (C, NCH_2_ CH_2_*C*H_2_CH_3_), 22.1 (C, NCH_2_*C*H_2_CH_2_CH_3_), 51.2 (C, *C*H_2_CH_2_CH_2_CH_3_), 111.2 (2CH, C-5, C-7), 120.9 (CH, C-4a), 126.0 (2C, C-3′, C-5′), 127.2 (CH, C-8), 130.6 (2C, C-2′, C-6′), 134.6 (C, C-6), 140.4 (C, C-8a), 142.3 (C, C-1′), 144.7 (C, C-4′), 155.0 (CO-C-4).

### 3.2. Procedure of Alpha-Glucosidase Inhibition Assay

Alpha-glucosidase inhibition activity was carried out by the Pierre *et al.* method with slight modifications, and alpha-Glucosidase (Cat No. 5003-1KU Type I) from *Saccharomyces cereviciae* was utilized for the experimental protocol due to its resemblances to the structure and function of yeast/mammalian enzyme. For test, saline phosphate buffer (70 μL, 50 mM with pH 6.8), alpha-glucosidase enzyme (10 μL, 0.0234 units) and test compound (10 μL, 0.5 mM) were taken in test tubes and incubated for 10 min at 37 °C. Absorbance of these prepared samples was recorded at 400 nm. 10 μL *p*-nitrophenyl-alpha-D-glucopyranoside (0.5 mM, ‘substrate’, code No. N1377 from Sigma) was used to initiate the reaction and test tubes were allowed to stand for 30 min. Then, the variation in concentration of free substrate was noted by the change in absorbance. The enzyme percentage inhibition was estimated by the following formula and IC_50_ values were calculated by ‘EZ-Fit enzyme kinetics software.

% *Inhibition* = [(*Abs. of test* − *Abs. of control*)/*Abs. of control*] × 100

## 4. Conclusions

A series of 1,2,3-benzotriazin-4(3*H*)-one based sulfonamides was designed, synthesized and discovered to be effective alpha-glucosidase inhibitors. Among all other derivatives, compound **12e** (IC_50_ 32.37 ± 0.15 µM) was found to be a better inhibitor as compared to the standard drug acarbose (IC_50_ 37.38 ± 0.12 µM). Moreover, the inhibition potential of hybrid **12f** (IC_50_ 37.75 ± 0.11 µM) was observed to be comparable to the reference drug. The enzyme inhibition mechanism was unlocked by molecular docking studies and significant interactions between the leading ligands and the targeted receptor were explored. In DFT, frontier molecular orbital analysis and molecular electrostatic potential studies were employed to understand various electronic parameters of all synthesized compounds. The least energy gap (E_gap_) value between HOMO and LUMO depicted the reactive nature of **12e** and **12f.** In addition, the higher electrophilicity value of these compounds also acknowledged their interactive nature towards biomolecules. The electrophilic and nucleophilic sites of all synthesized compounds were shown by MEP studies. Hence, the *in vitro* alpha-glucosidase inhibitory results were in good agreement with the outcomes of molecular docking and the performed DFT calculations.

## Figures and Tables

**Table 1:** Results of alpha-glucosidase inhibition studies.

Sr. No.	Codes	Structures	% Inhibition at 0.5 mM	IC_50_ (μM)
1.	**11a**	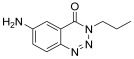	26.12 ± 0.13	ND
2.	**11b**	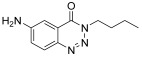	27.12 ± 0.13	ND
3.	**12a**	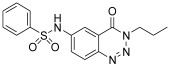	51.25 ± 0.16	˂500
4.	**12b**	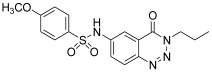	91.73 ± 0.17	39.21 ± 0.12
5.	**12c**	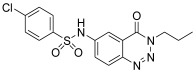	78.12 ± 0.16	53.76 ± 0.14
6.	**12d**	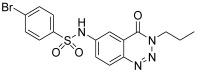	79.42 ± 0.15	55.21 ± 0.12
7.	**12e**	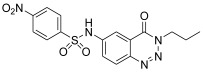	96.27 ± 0.19	32.37 ± 0.15
8.	**12f**	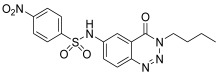	93.27 ± 0.14	37.75 ± 0.11
9.	**Standard**	acarbose	92.23 ± 0.16	37.38 ± 0.12

**Table 2:** Electronic parameters of synthesized compounds (**12a–f**).

Compounds	E_HOMO_	E_LUMO_	E_gap_	I	A	η	S	μ	Χ	ω	D
**12a**	−6.62	−2.06	4.55	6.62	2.06	2.28	0.22	4.34	4.34	4.14	5.54
**12b**	−6.52	−1.98	4.54	6.52	1.98	2.27	0.22	4.25	4.25	3.98	7.07
**12c**	−6.73	−2.17	4.56	6.73	2.17	2.28	0.22	4.45	4.45	4.34	3.67
**12d**	−6.70	−2.15	4.55	6.70	2.15	2.27	0.22	4.43	4.43	4.31	3.89
**12e**	−7.02	−3.33	3.70	7.02	3.33	1.85	0.27	5.18	5.18	7.25	2.30
**12f**	−7.01	−3.33	3.68	7.01	3.33	1.84	0.27	5.17	5.17	7.26	2.35

**Table 3:** Physiochemical properties of all synthesized compounds **7–12f**.

Entry	Compound	Molecular Formula	Molecular Weight	Physical Appearance	% Yield	m.p. (℃)	Solubility
1.	**7**	C_8_H_5_NO_3_	163.13	Light yellow powder	85%	230–232	Ethanol, DMSO
2.	**8**	C_8_H_4_N_2_O_5_	208.13	Yellow powder	62%	219–221	Ethanol, DMSO
3.	**9a**	C_10_H_13_N_3_O_3_	223.23	Mustard powder	60%	195–197	Ethanol, DMSO
4.	**9b**	C_11_H_15_N_3_O_3_	237.26	Mustard powder	58%	197–199	Ethanol, DMSO
5.	**10a**	C_10_H_10_N_4_O_3_	234.21	Light yellow powder	92%	215–217	Ethanol, DMSO
6.	**10b**	C_11_H_12_N_4_O_3_	248.24	White powder	91%	217–218	Ethanol, DMSO
7.	**11a**	C_10_H_12_N_4_O	204.23	Transparent crystals	80%	185–186	Methanol, DMSO
8.	**11b**	C_11_H_14_N_4_O	218.26	Transparent crystals	72%	191–192	Methanol, DMSO
9.	**12a**	C_16_H_16_N_4_O_3_S	344.39	Brown powder	82%	171–173	Ethanol, DMSO
10.	**12b**	C_17_H_18_N_4_O_4_S	374.41	Brown powder	75%	175–177	Ethanol, DMSO
11.	**12c**	C_16_H_15_ClN_4_O_3_S	378.83	Brown powder	68%	169–171	Ethanol, DMSO
12.	**12d**	C_16_H_15_BrN_4_O_3_S	423.28	Brown powder	78%	171–179	Ethanol, DMSO
13.	**12e**	C_16_H_15_N_5_O_5_S	389.39	Brown powder	71%	167–169	Ethanol, DMSO
14.	**12f**	C_17_H_17_N_5_O_5_S	403.41	Brown powder	62%	179–181	Ethanol, DMSO

## Data Availability

The data presented in this study are available in the Supplementary Materials. Samples of the compounds are available from the authors.

## Supplementary Materials

The following supporting information can be downloaded at: https://www.mdpi.com/article/10.3390/molecules27206783/s1, Figure S1: FTIR spectrum of 7; Figure S2: FTIR spectrum of 8; Figure S3: FTIR spectrum of 9a; Figure S4: FTIR spectrum of 9b; Figure S5: FTIR spectrum of 10a; Figure S6: FTIR spectrum of 10b; Figure S7: FTIR spectrum of 11a; Figure S8: 1H NMR spectrum of 11a; Figure S9: 13C NMR spectrum of 11a; Figure S10: FTIR spectrum of 11b; Figure S11: 1H-NMR spectrum of 11b; Figure S12: 13C-NMR spectrum of 11b; Figure S13: FTIR spectrum of 12a; Figure S14: 1H-NMR spectrum of 12a; Figure S15: 13C-NMR spectrum of 12a; Figure S16: FTIR spectrum of 12b; Figure S17: 1H-NMR spectrum of 12b; Figure S18: 13C-NMR spectrum of 12b; Figure S19: FTIR spectrum of 12c; Figure S20: 1H-NMR spectrum of 12c; Figure S21: 13C-NMR spectrum of 12c; Figure S22: FTIR spectrum of 12d; Figure S23: 1H-NMR spec-trum of 12d; Figure S24: 13C-NMR spectrum of 12d; Figure S25: FTIR spectrum of 12e; Fig-ure S26: 1H-NMR spectrum of 12e; Figure S27: 13C-NMR spectrum of 12e; Figure S28: FTIR spectrum of 12f; Figure S29: 1H-NMR spectrum of 12f; Figure S30: 13C-NMR spectrum of 12f; Table S1:Binding energies of the docked compounds 7a-f inside the active sites of α-glucosidase.
