# Supplementary material for: Experimental and Computational Analysis of Newly Synthesized Benzotriazinone Sulfonamides as Alpha-Glucosidase Inhibitors
Source: Molecules. 2022 Oct 11;27(20):6783. doi: 10.3390/molecules27206783 (PMC9612054; doi:10.3390/molecules27206783)
Supplement: Supplementary file 1 [file molecules-27-06783-s001.zip › molecules-1920268-supplementary.pdf]

Experimental and Computational Analysis of Newly Synthesized Benzotriazinone Sulfonamides as Alpha-Glucosidase Inhibitors

Zunera Khalid [1], Maha Abdallah Alnuwaiser [2], Hafiz Adnan Ahmad [3], Syed Salman Shafqat [4,*], Munawar Ali Munawar [3,5], Kashif Kamran [6], Muhammad Mujtaba Abbas [7], M.A. Kalam [8] and Menna A. Ewida [9]

[1] Department of Chemistry, Kinnaird College for Women, Lahore 54000, Pakistan
[2] Department of Chemistry, College of Science, Princess Nourah bint Abdulrahman University, P.O. Box 84428, Riyadh 11671, Saudi Arabia
[3] School of Chemistry, University of the Punjab, Lahore 54590, Pakistan
[4] Department of Chemistry, Division of Science and Technology, University of Education, Lahore 54770, Pakistan
[5] Department of Basic and Applied Chemistry, FAST, University of Central Punjab, Lahore 54000, Pakistan
[6] Department of Physics, University of Agriculture, Faisalabad 38040, Pakistan
[7] Department of Mechanical Engineering, University of Engineering and Technology (New Campus), Lahore 54890, Pakistan
[8] Faculty of Engineering and IT, University of Technology, Sydney 2007, Australia
[9] Department of Pharmaceutical Chemistry, Faculty of Pharmacy, Future University in Egypt, New Cairo 11835, Egypt
* Correspondence: salman.shafqat@ue.edu.pk; Tel.: +92-331-413-9585

1. Supplementary Data

All ^{1}H-NMR and ^{13}C-NMR spectra were recorded in deuterated chloroform ($CDCl_3$) and dimethyl sulfoxide (DMSO-*d6*). Tetramethylsilane (TMS) was taken as an internal standard. In DMSO-*d6*, the solvent peak appeared at 2.49 ppm and the water peak was found at 3.47 ppm. The solvent peak of deuterated chloroform ($CDCl_3$) was observed at 7.24 ppm.

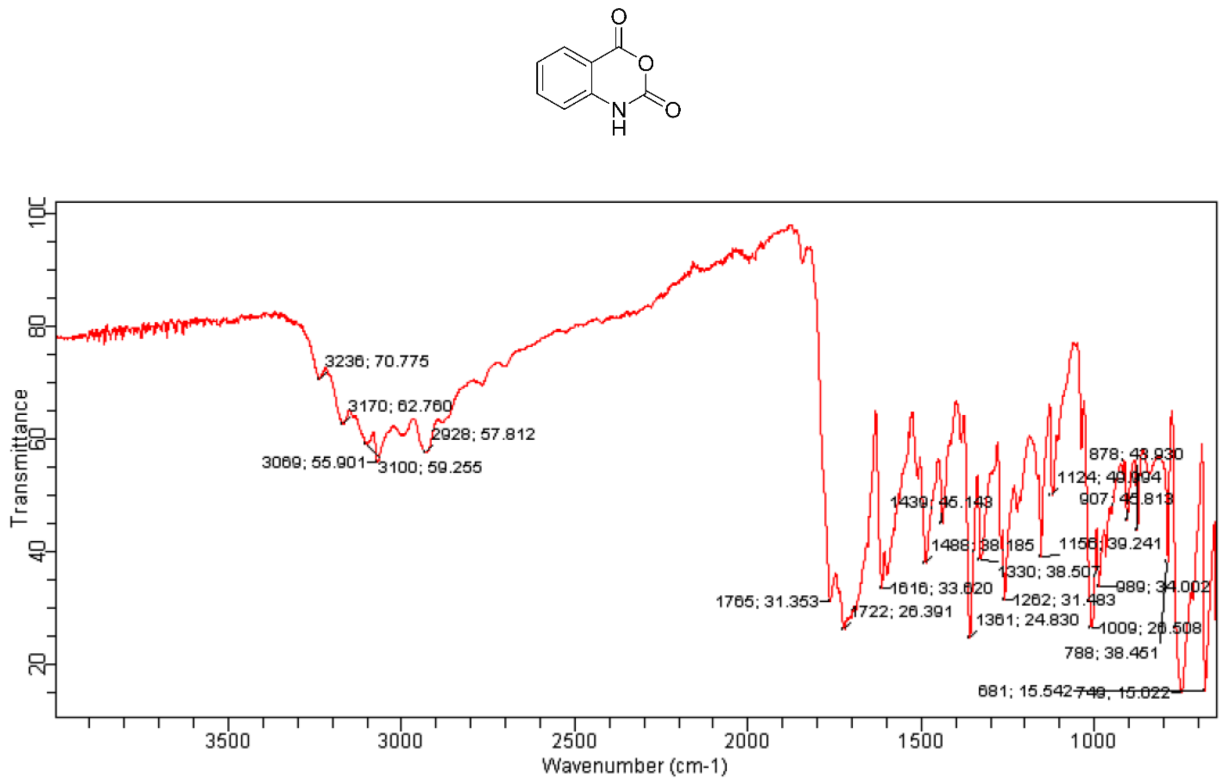

Figure S1. FTIR spectrum of **7**.

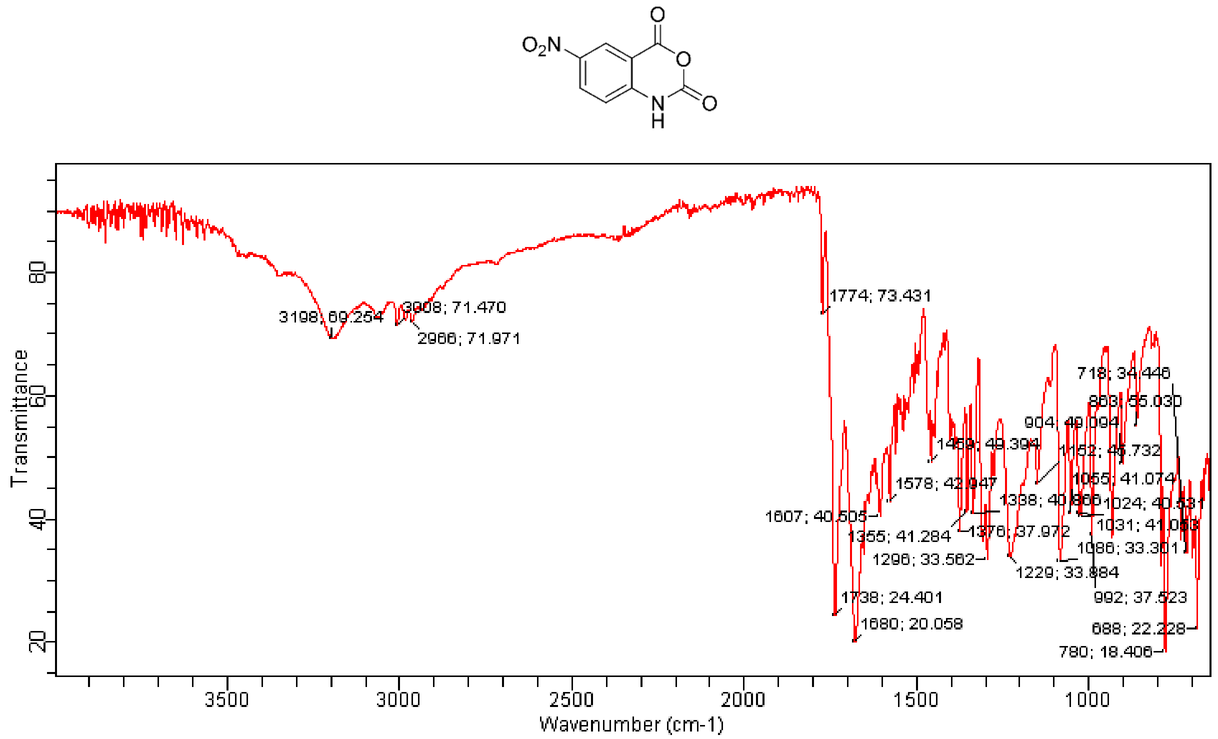

Figure S2. FTIR spectrum of **8**.

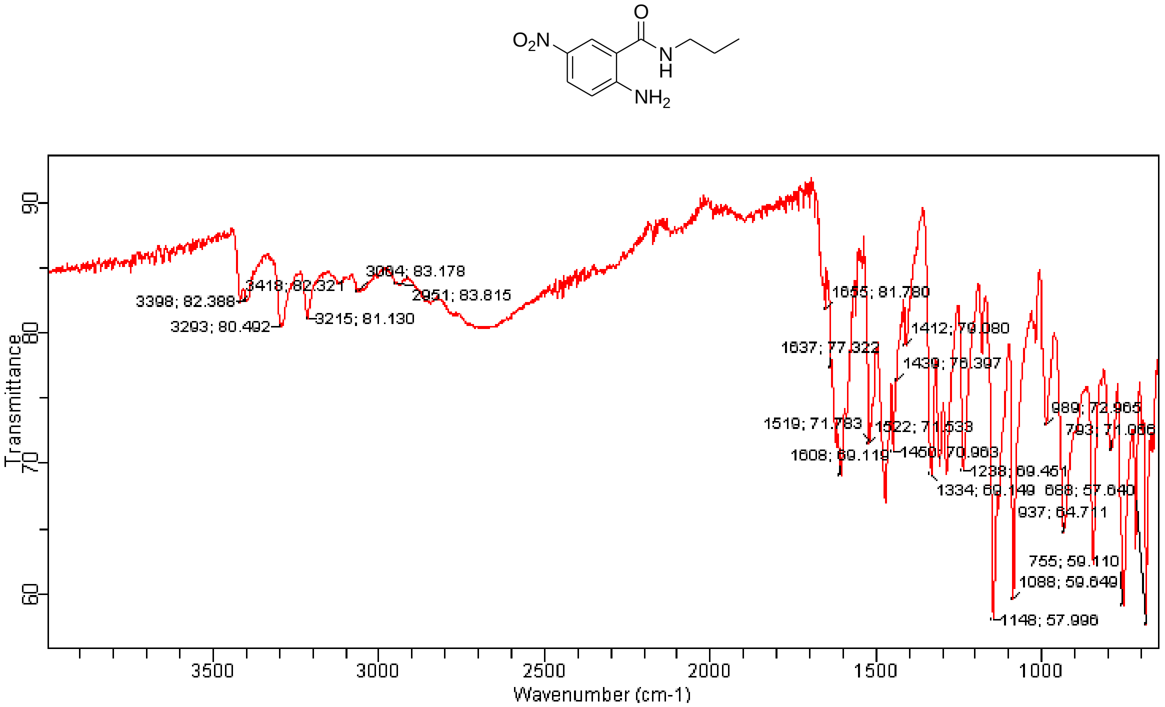

Figure S3. FTIR spectrum of **9a**.

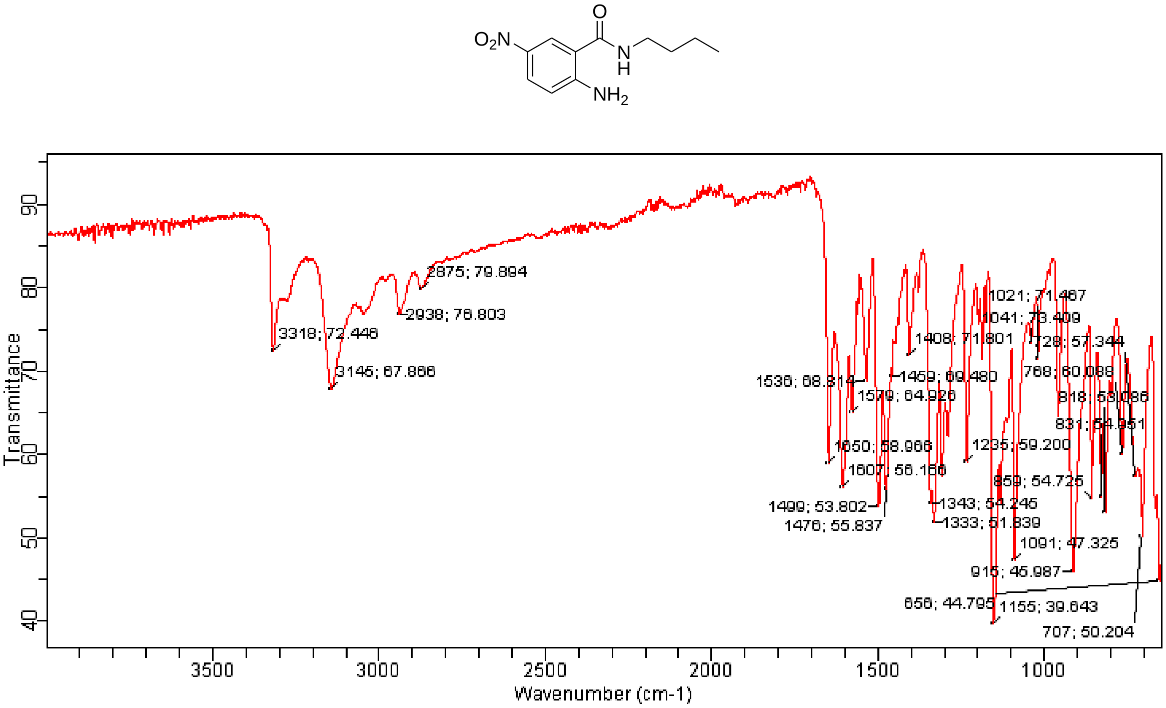

Figure S4. FTIR spectrum of **9b**.

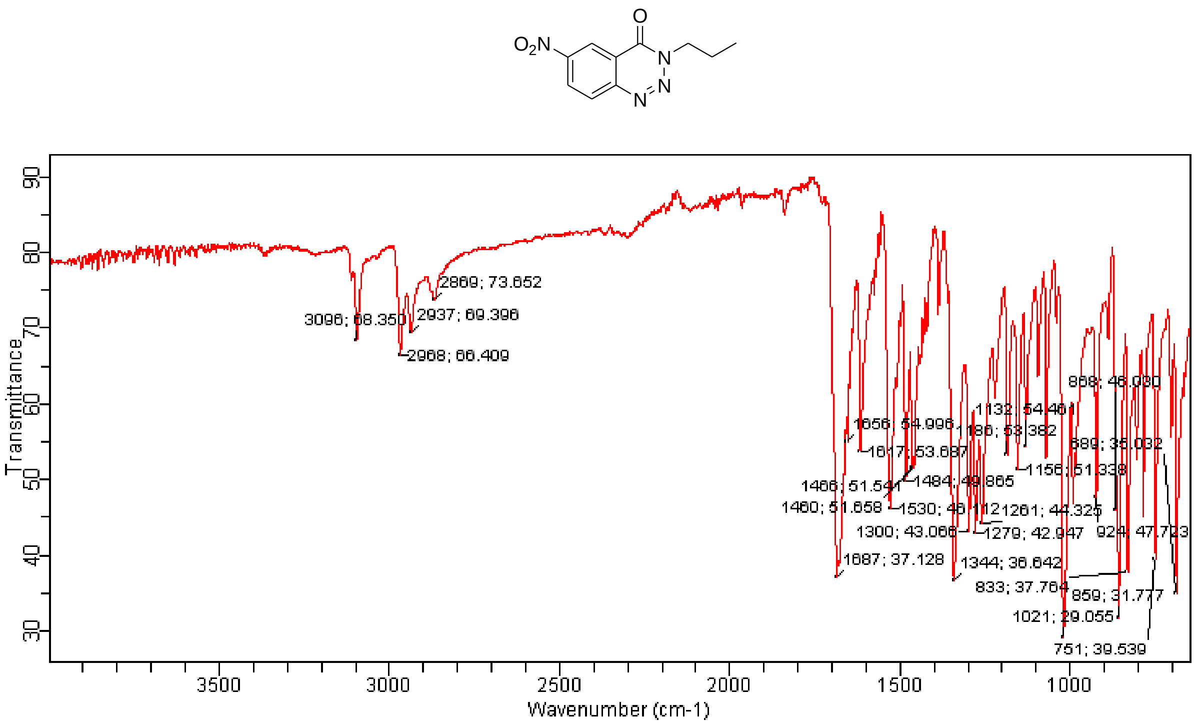

Figure S5. FTIR spectrum of **10a**.

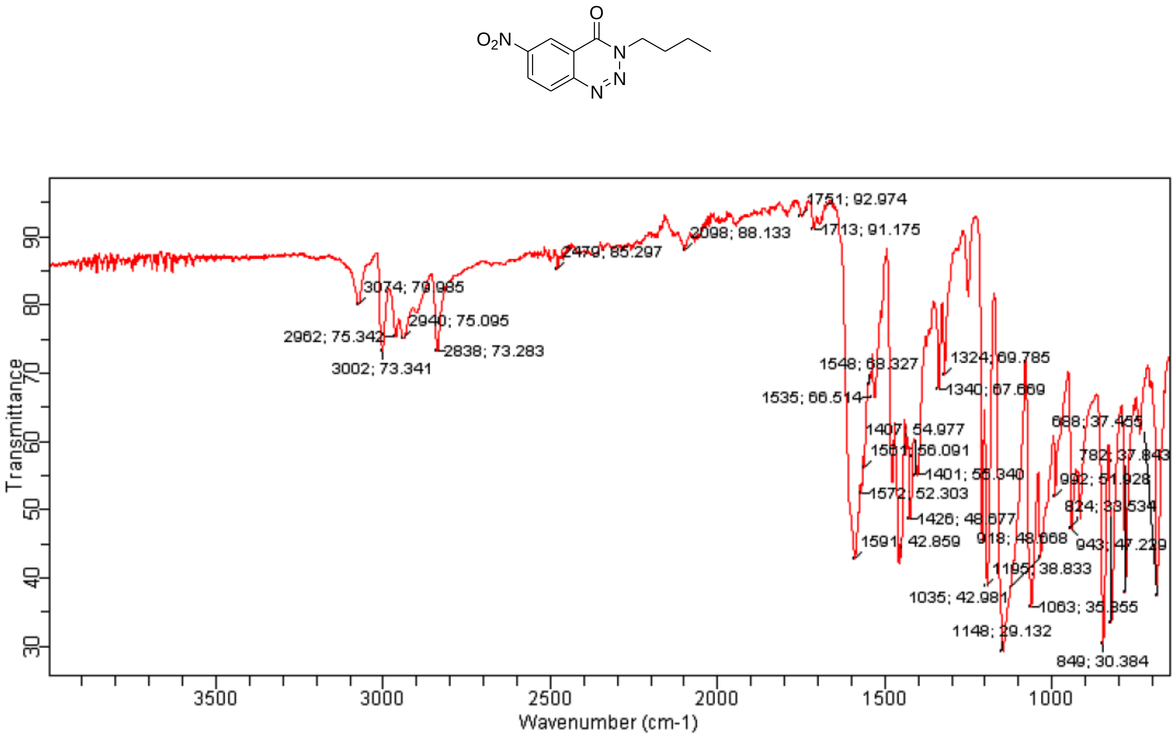

Figure S6. FTIR spectrum of **10b**.

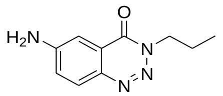

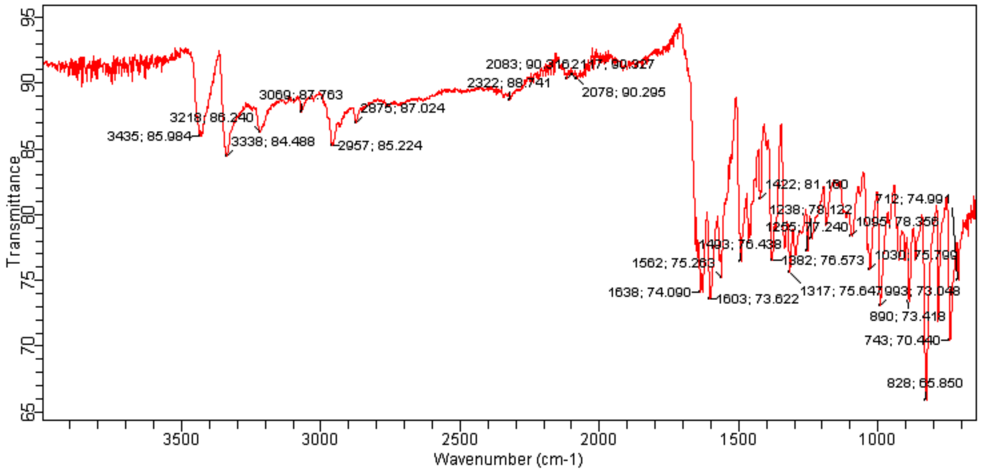

Figure S7. FTIR spectrum of **11a**.

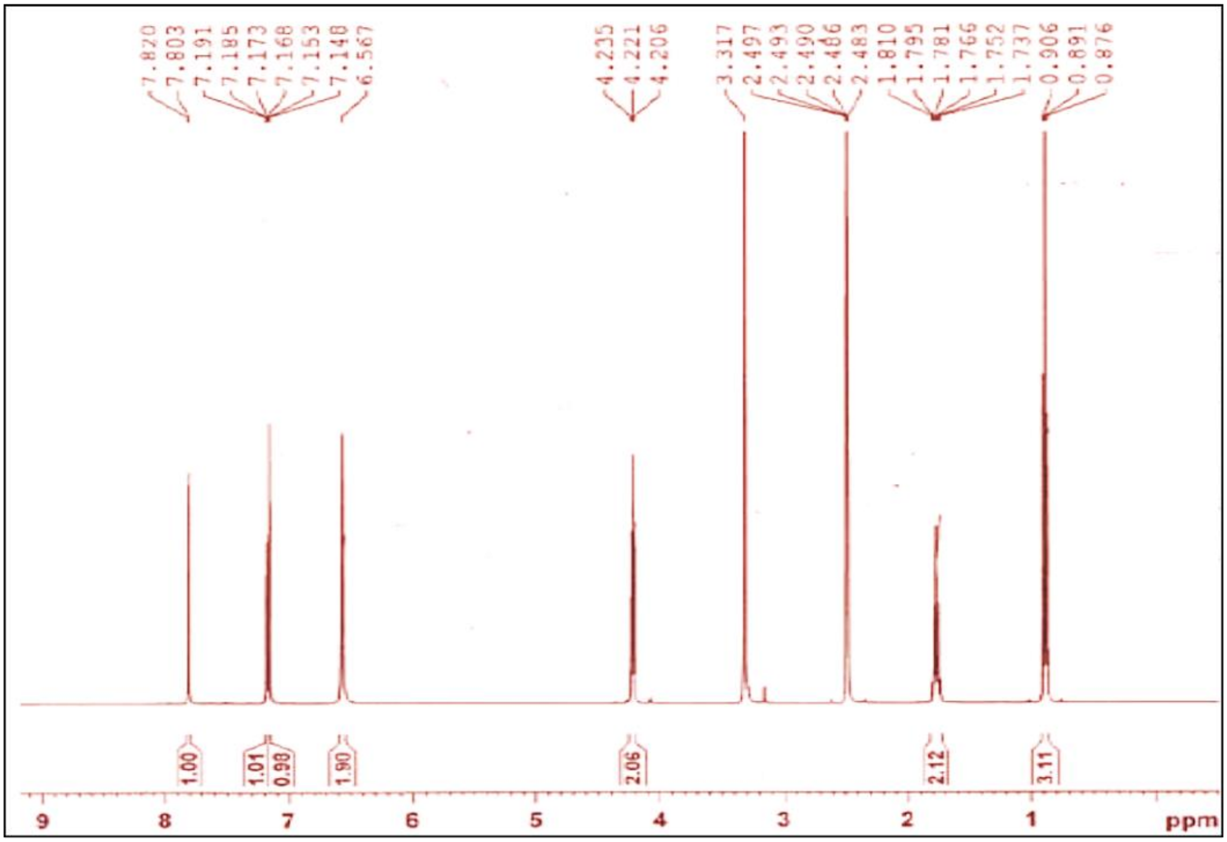

Figure S8. ^{1}H NMR spectrum of **11a**.

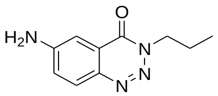

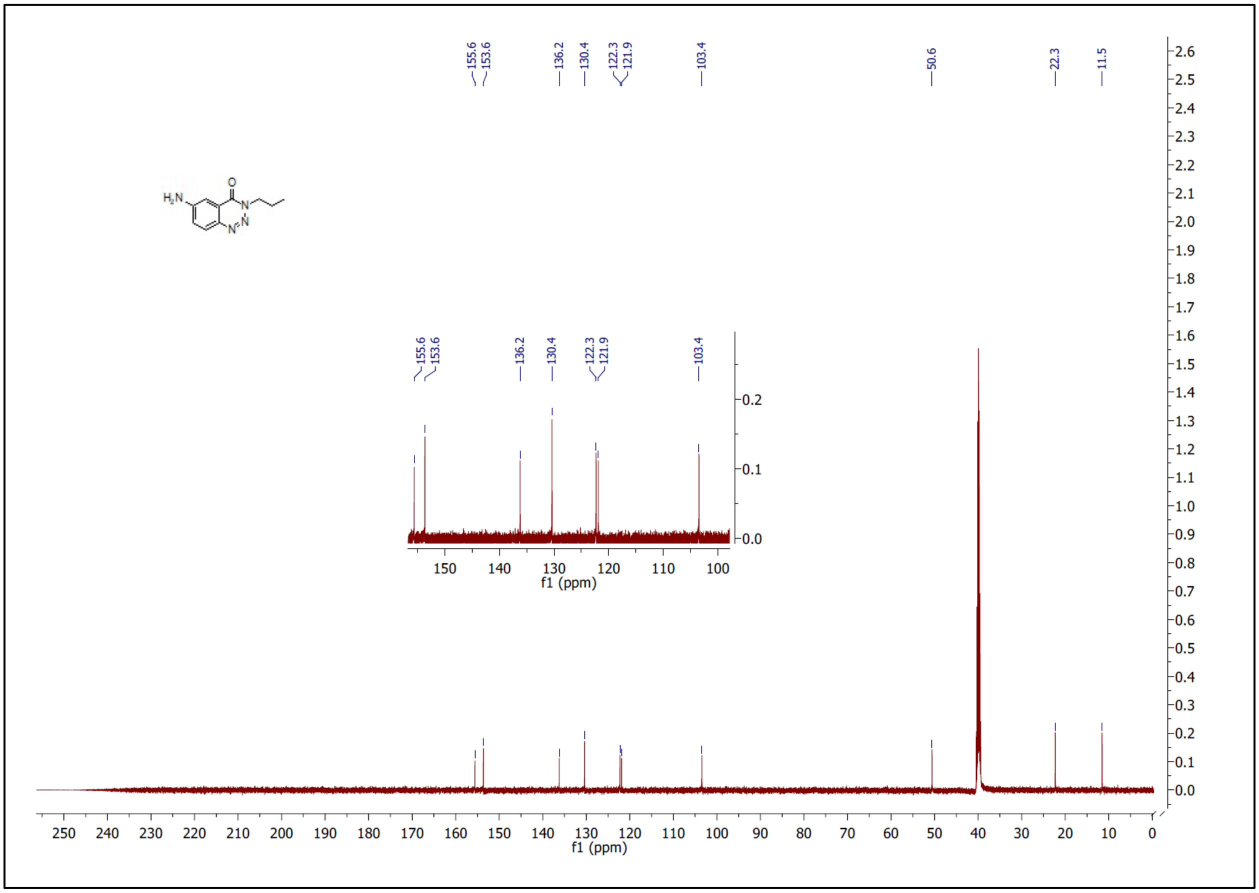

Figure S9. ^{13}C NMR spectrum of **11a**.

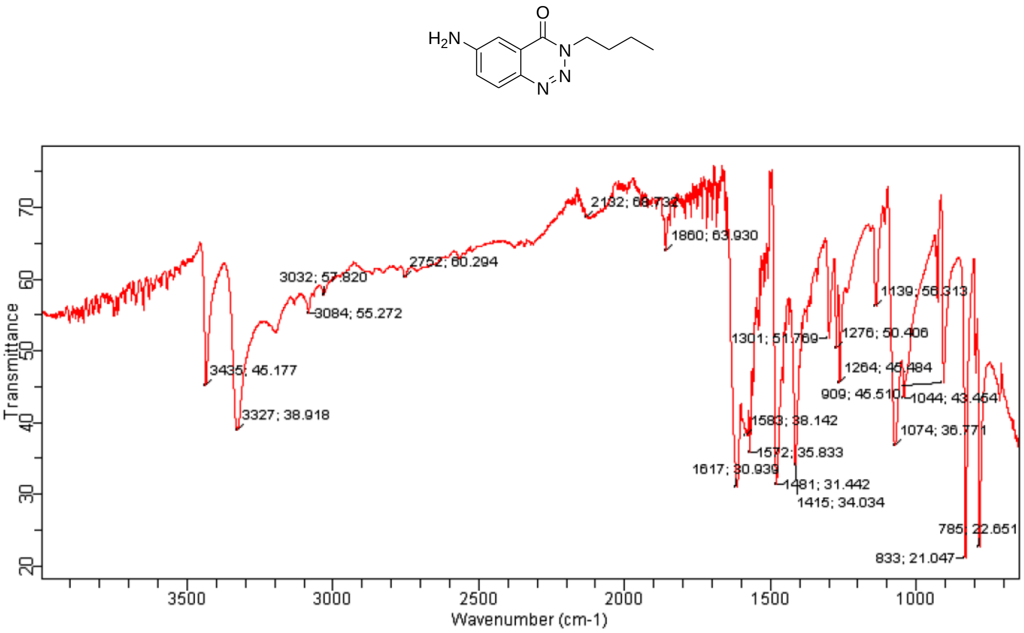

Figure S10. FTIR spectrum of **11b**.

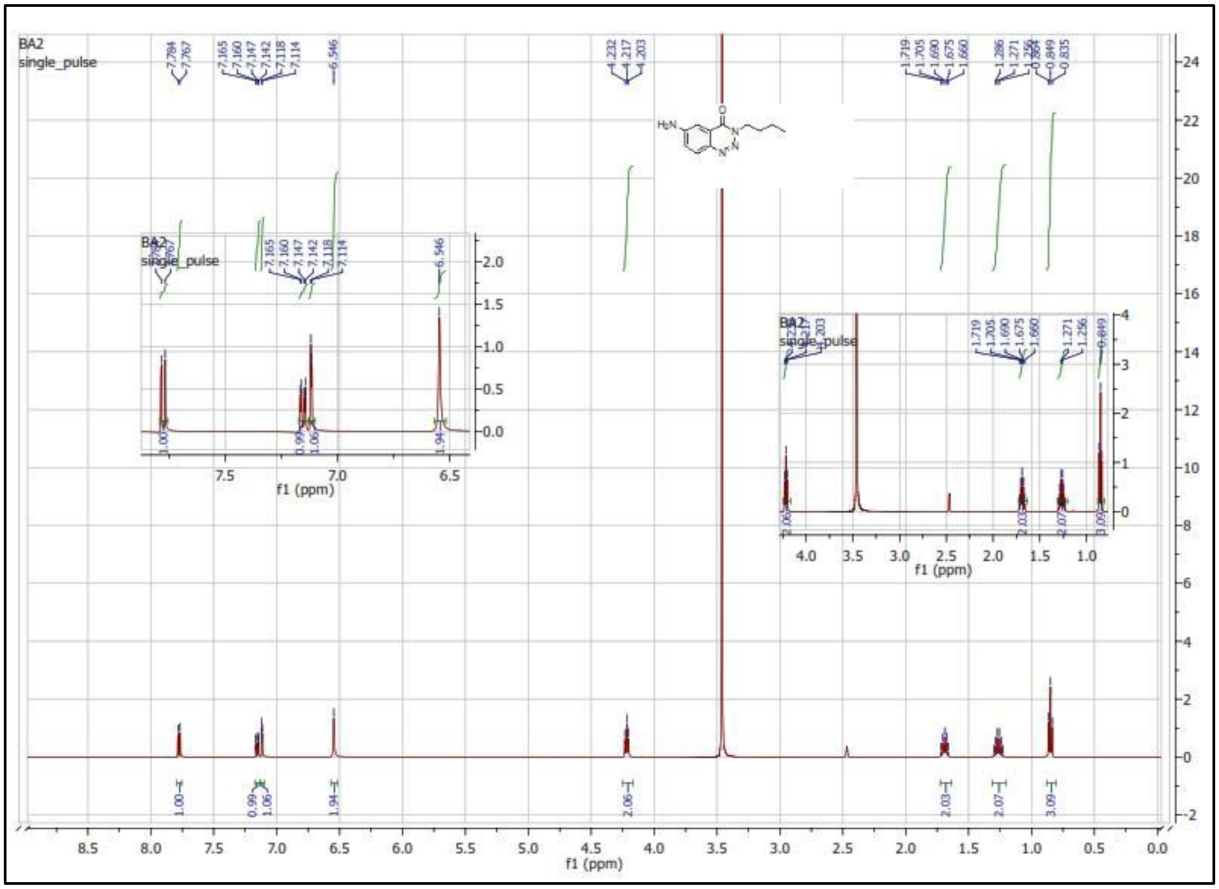

Figure S11. ^{1}H-NMR spectrum of **11b**.

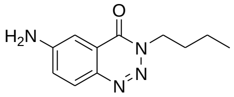

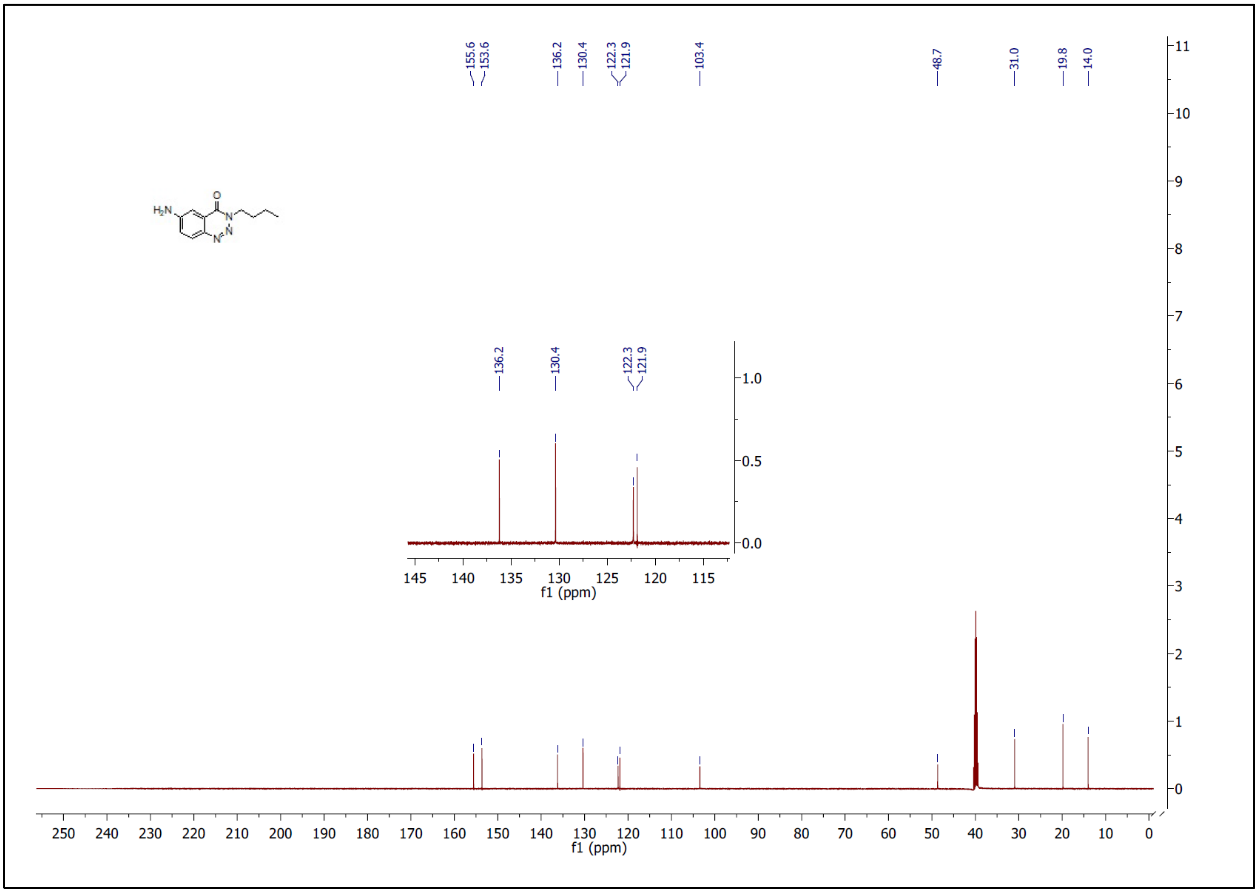

Figure S12. ^{13}C-NMR spectrum of **11b**.

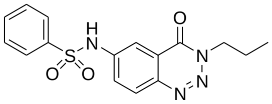

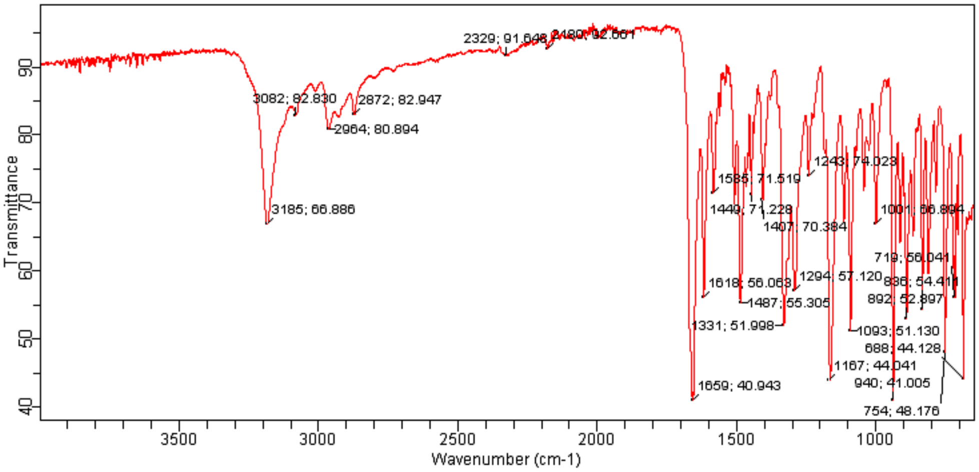

Figure S13. FTIR spectrum of **12a**.

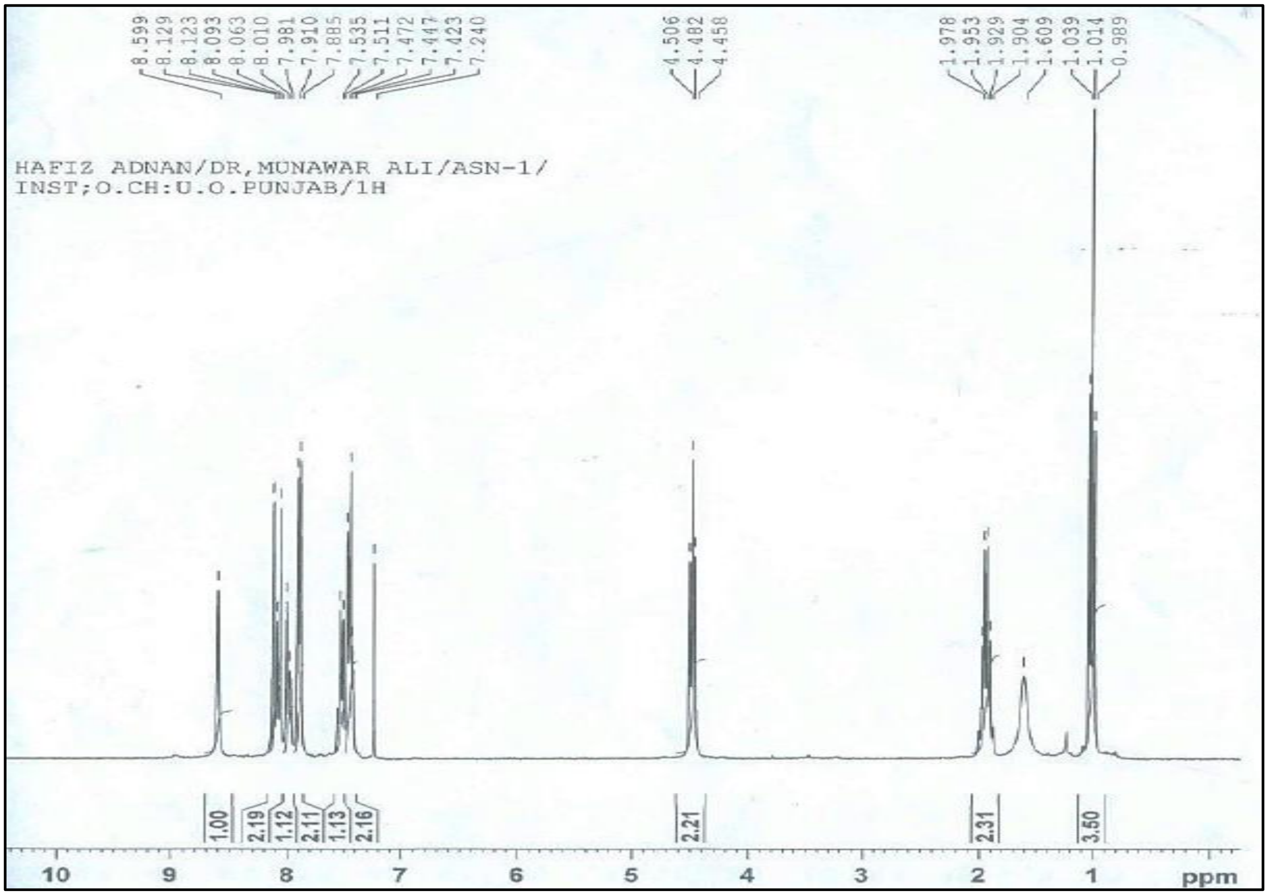

Figure S14. ^{1}H-NMR spectrum of **12a**.

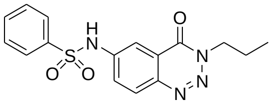

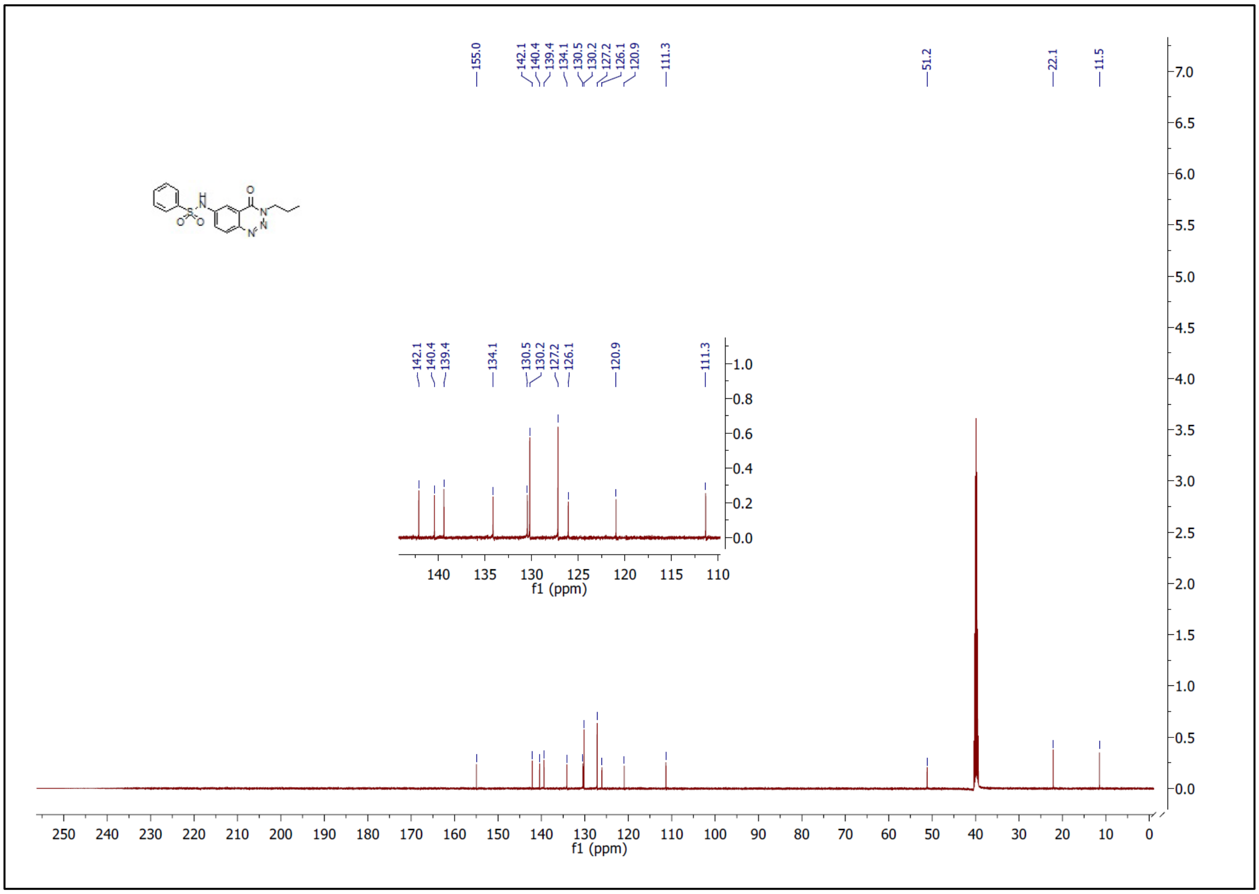

Figure S15: ^{13}C-NMR spectrum of **12a**.

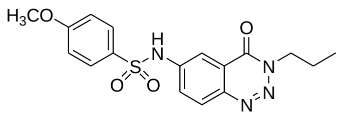

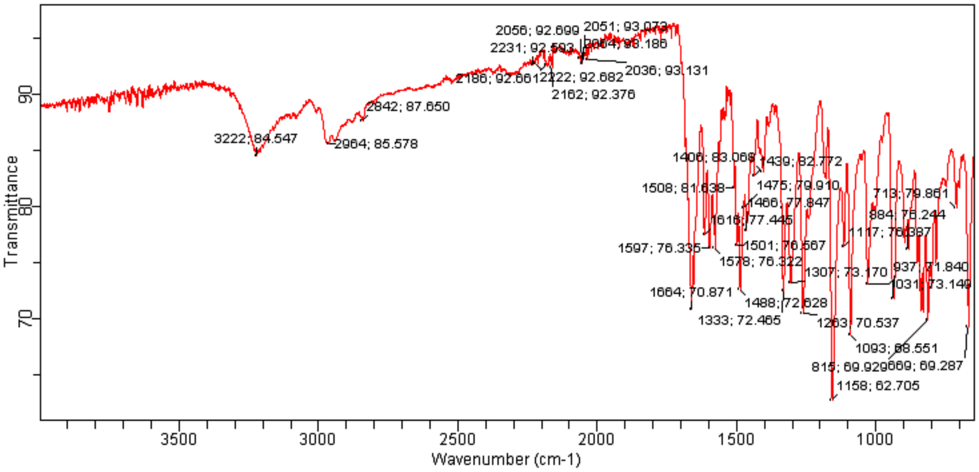

Figure S16: FTIR spectrum of **12b**.

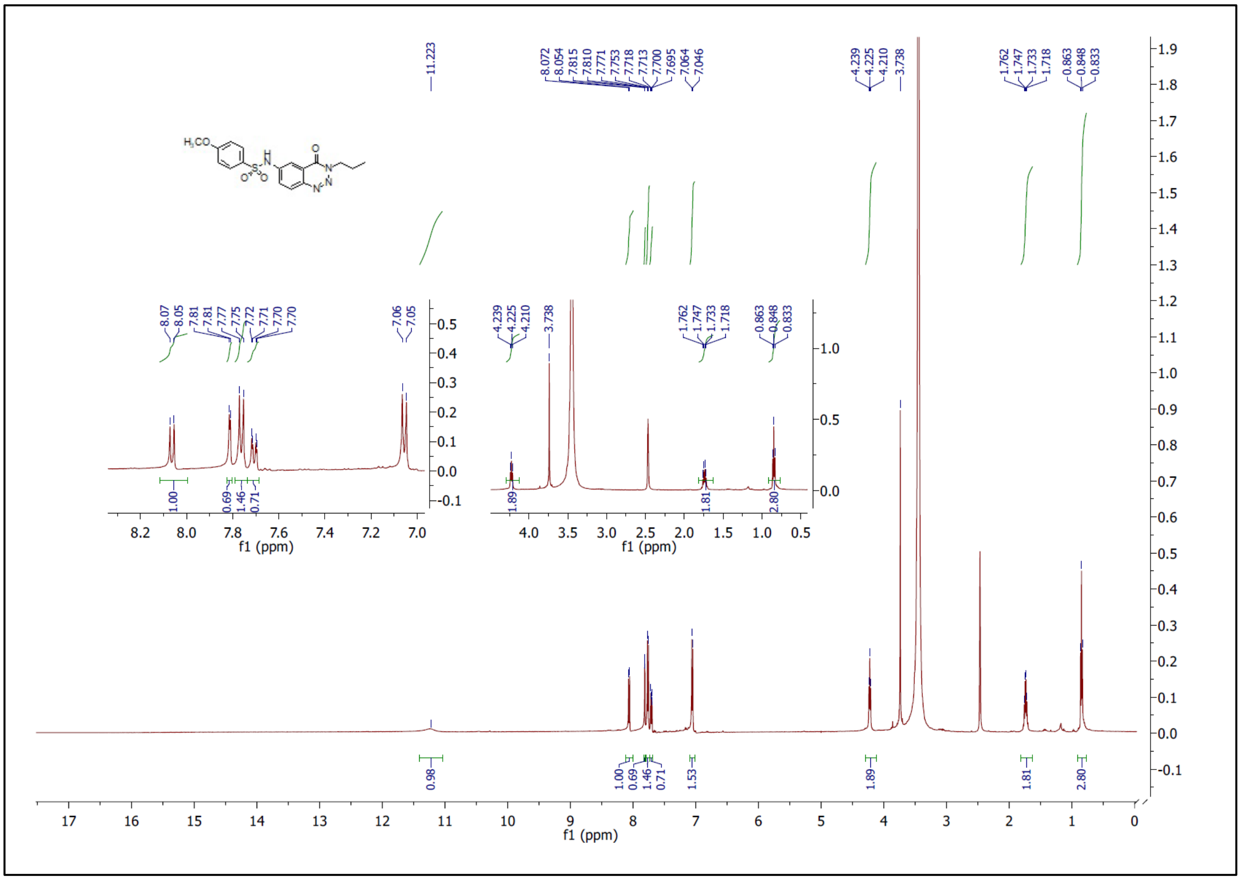

Figure S17. ^{1}H-NMR spectrum of **12b**.

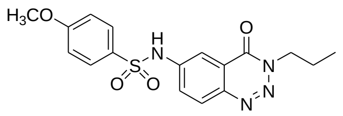

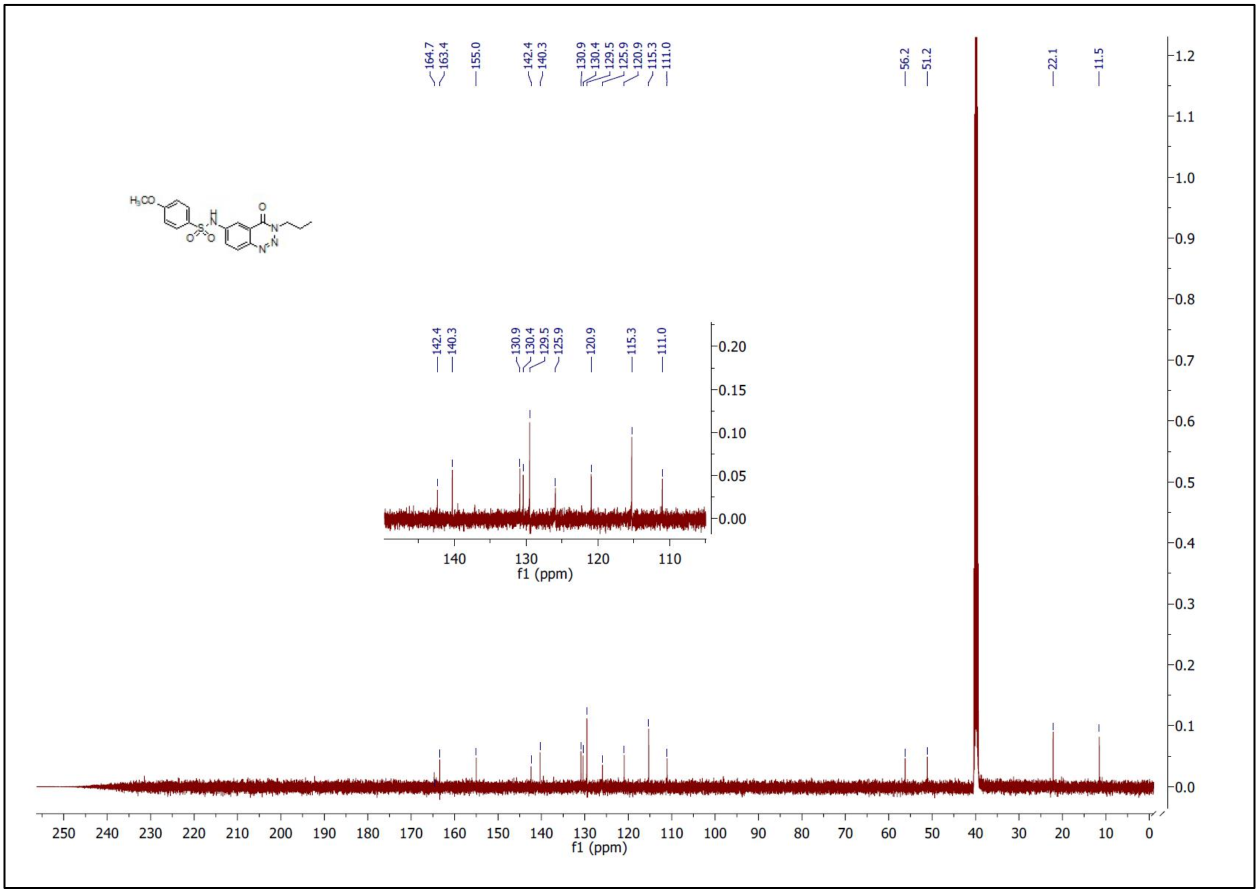

Figure S18. ^{13}C-NMR spectrum of **12b**.

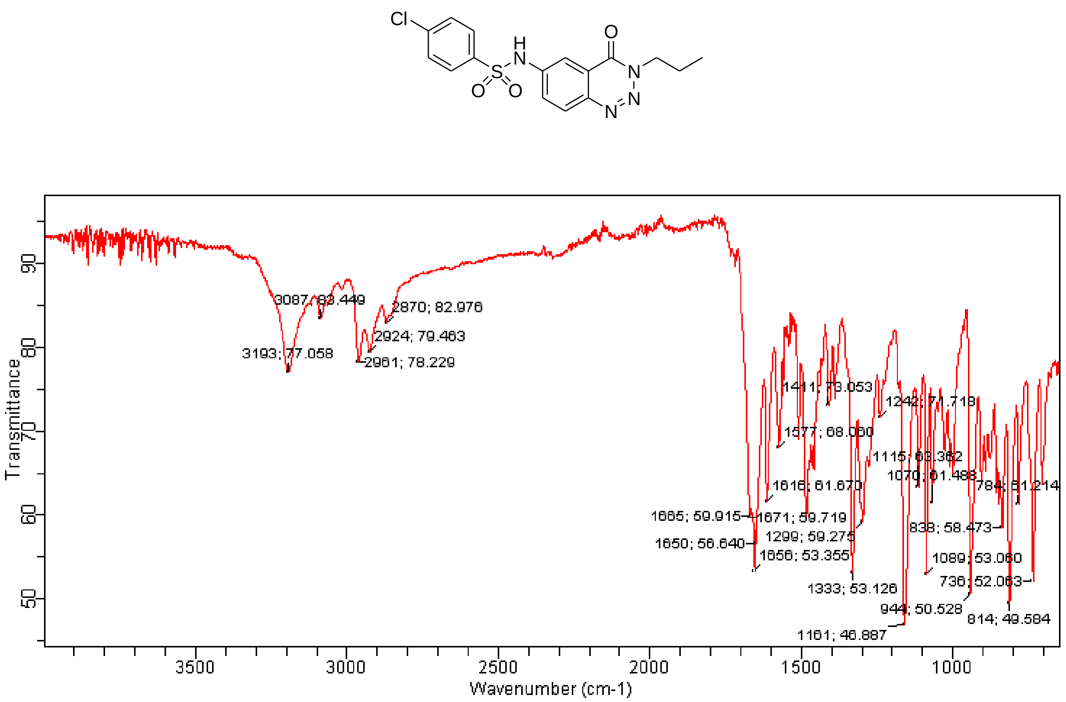

Figure S19. FTIR spectrum of **12c**.

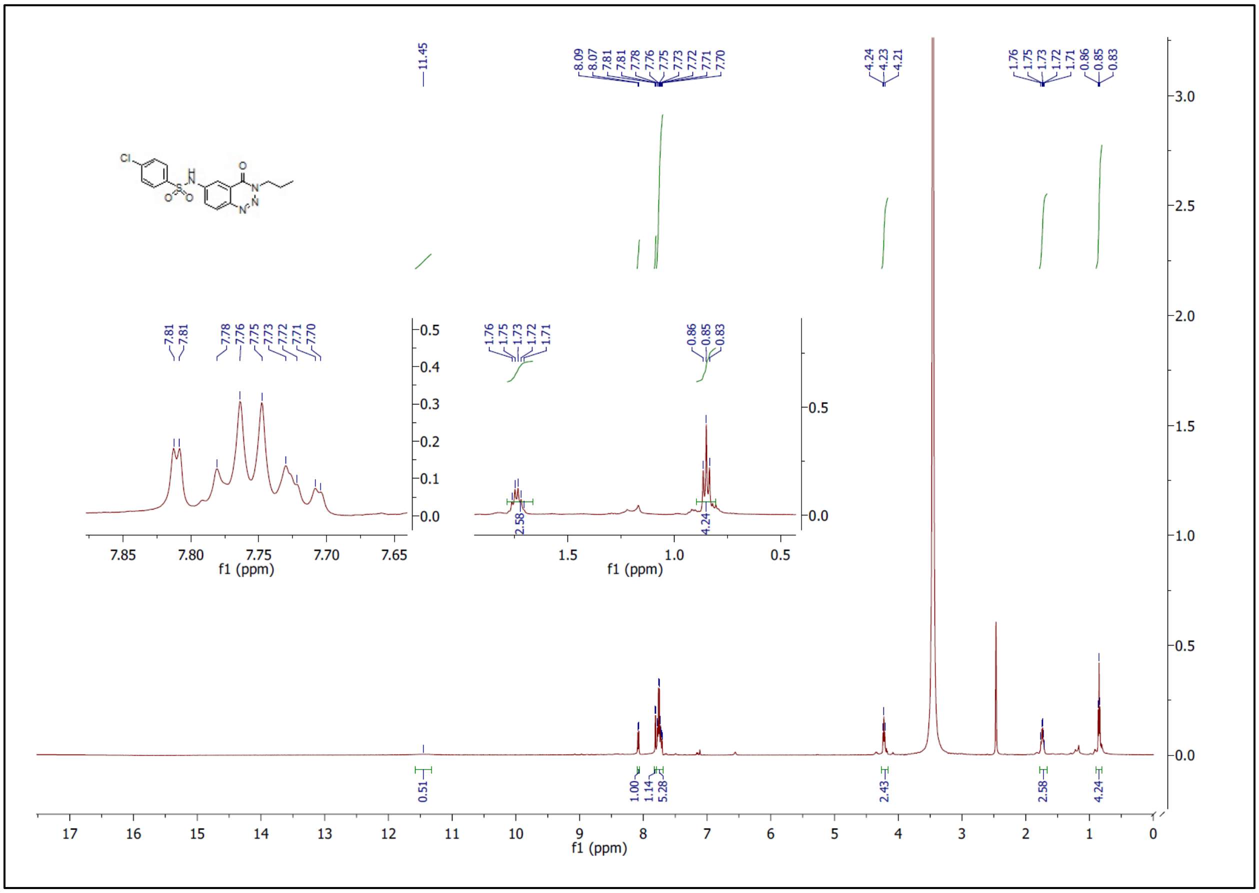

Figure S20. ^{1}H-NMR spectrum of **12c**.

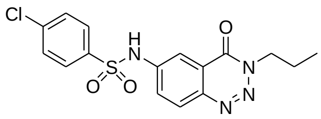

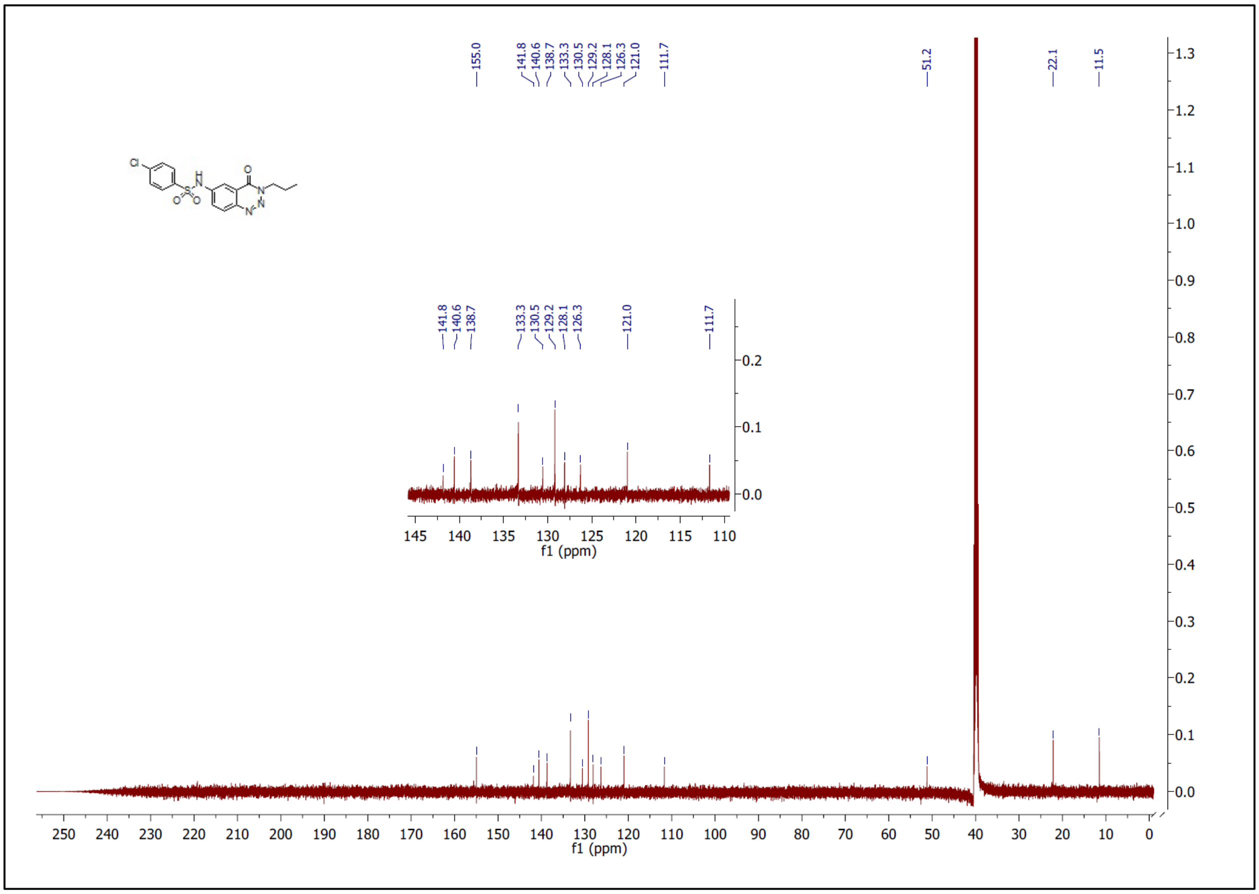

Figure S21. ^{13}C-NMR spectrum of **12c**.

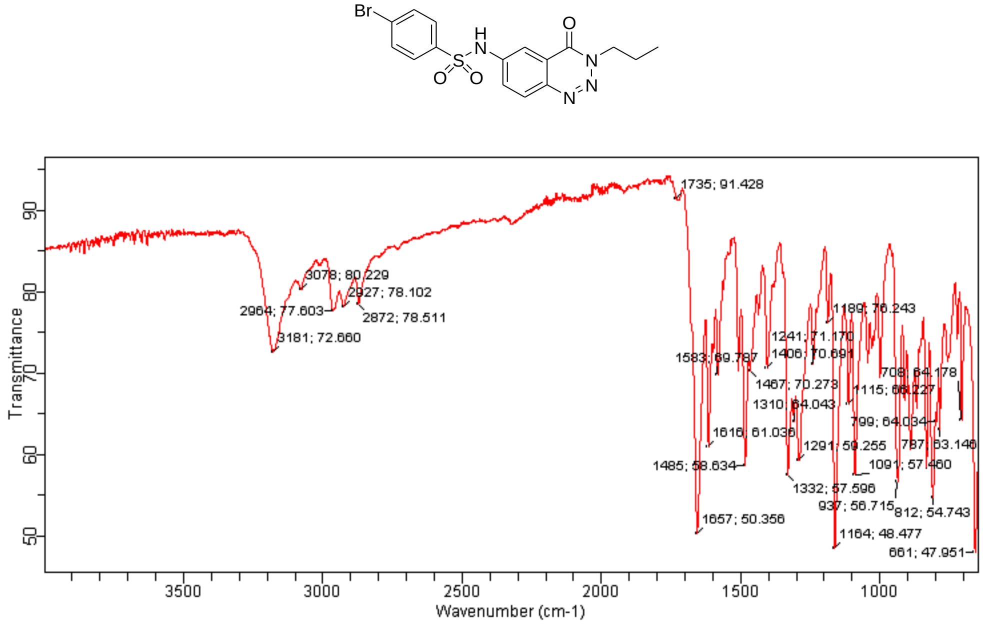

Figure S22. FTIR spectrum of **12d**.

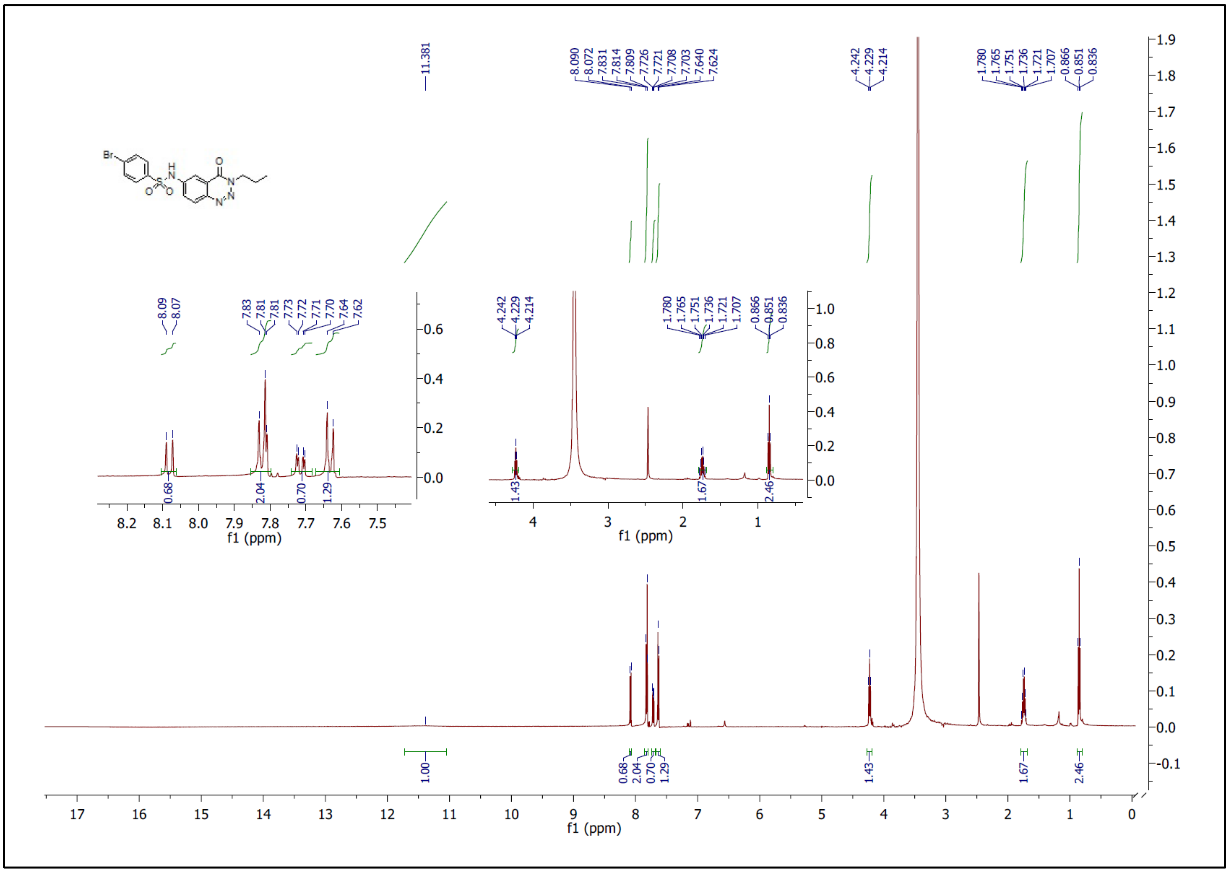

Figure S23. ^{1}H-NMR spectrum of **12d**.

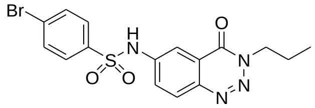

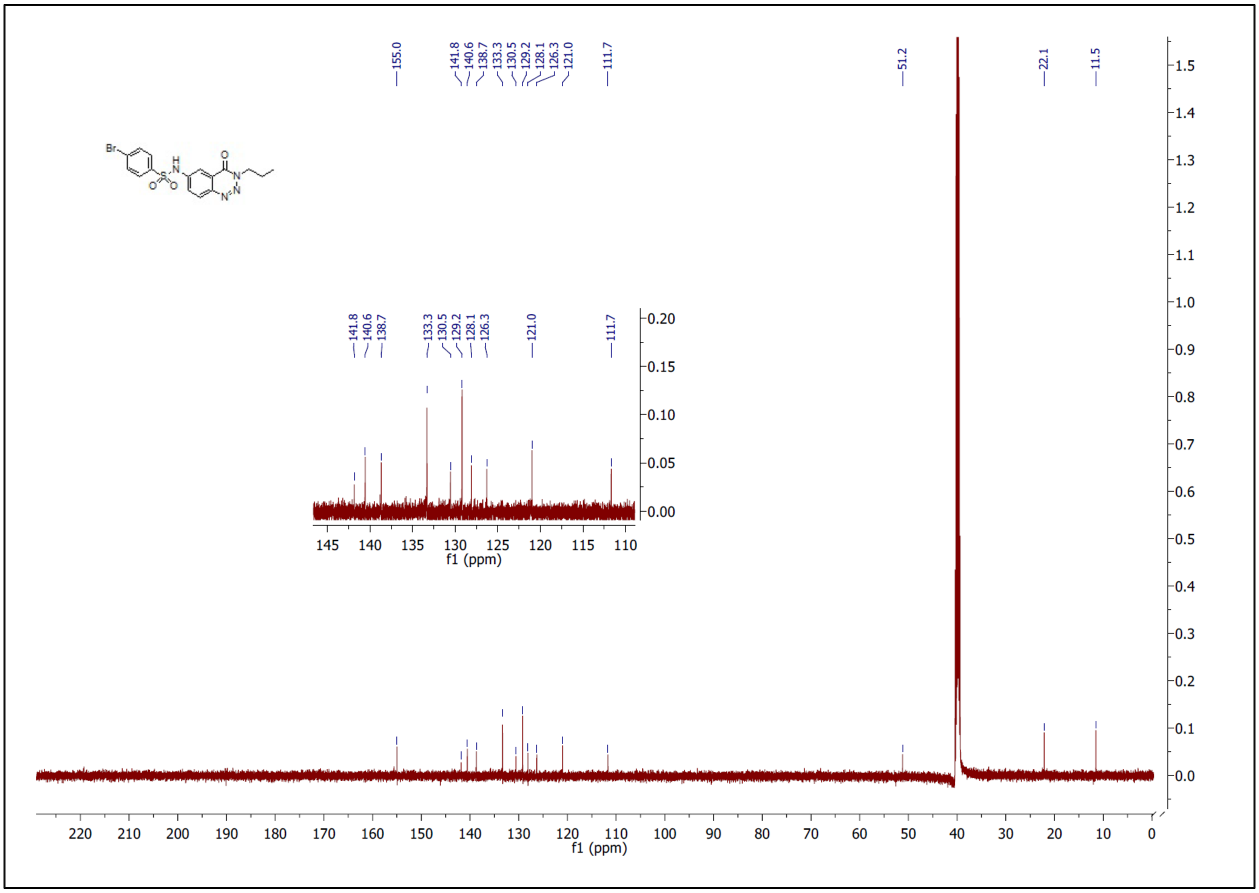

Figure S24. ^{13}C-NMR spectrum of **12d**.

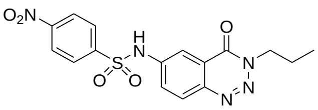

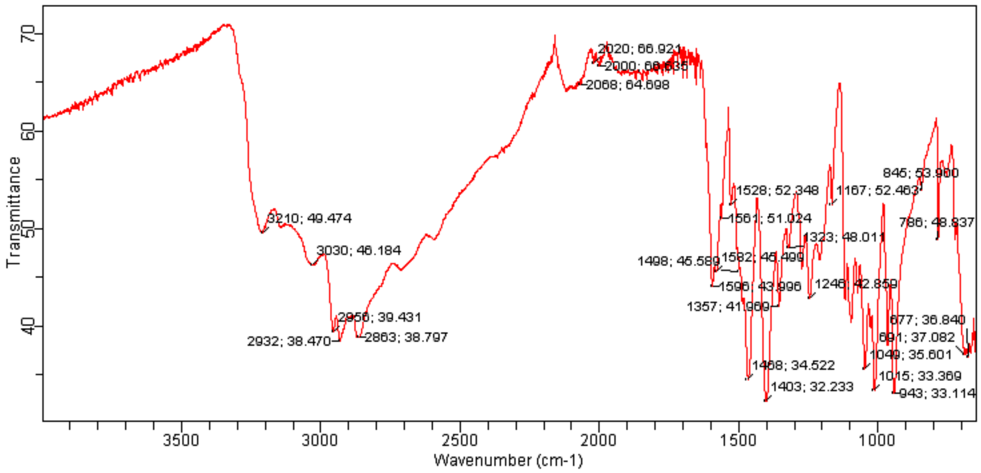

Figure S25. FTIR spectrum of **12e**.

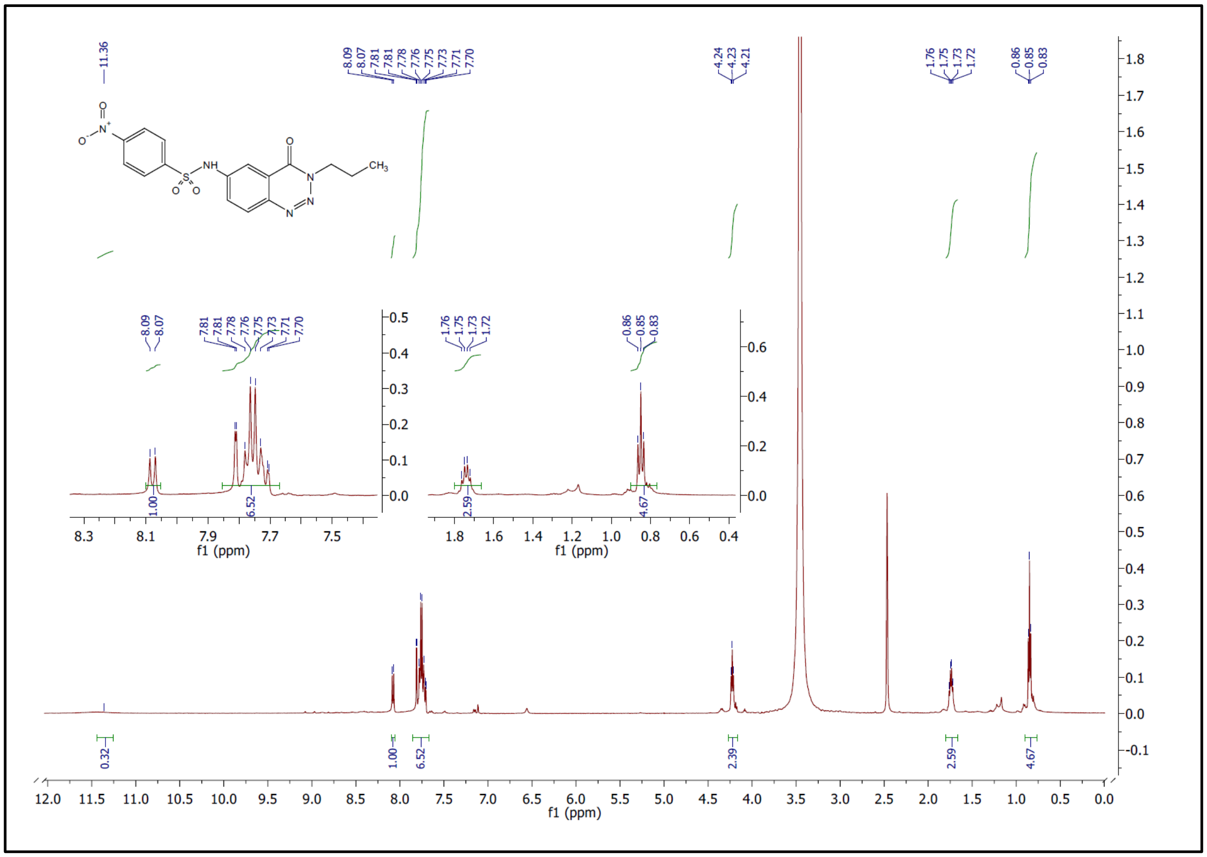

Figure S26. ^{1}H-NMR spectrum of **12e**.

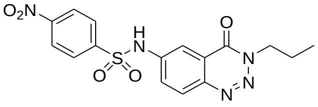

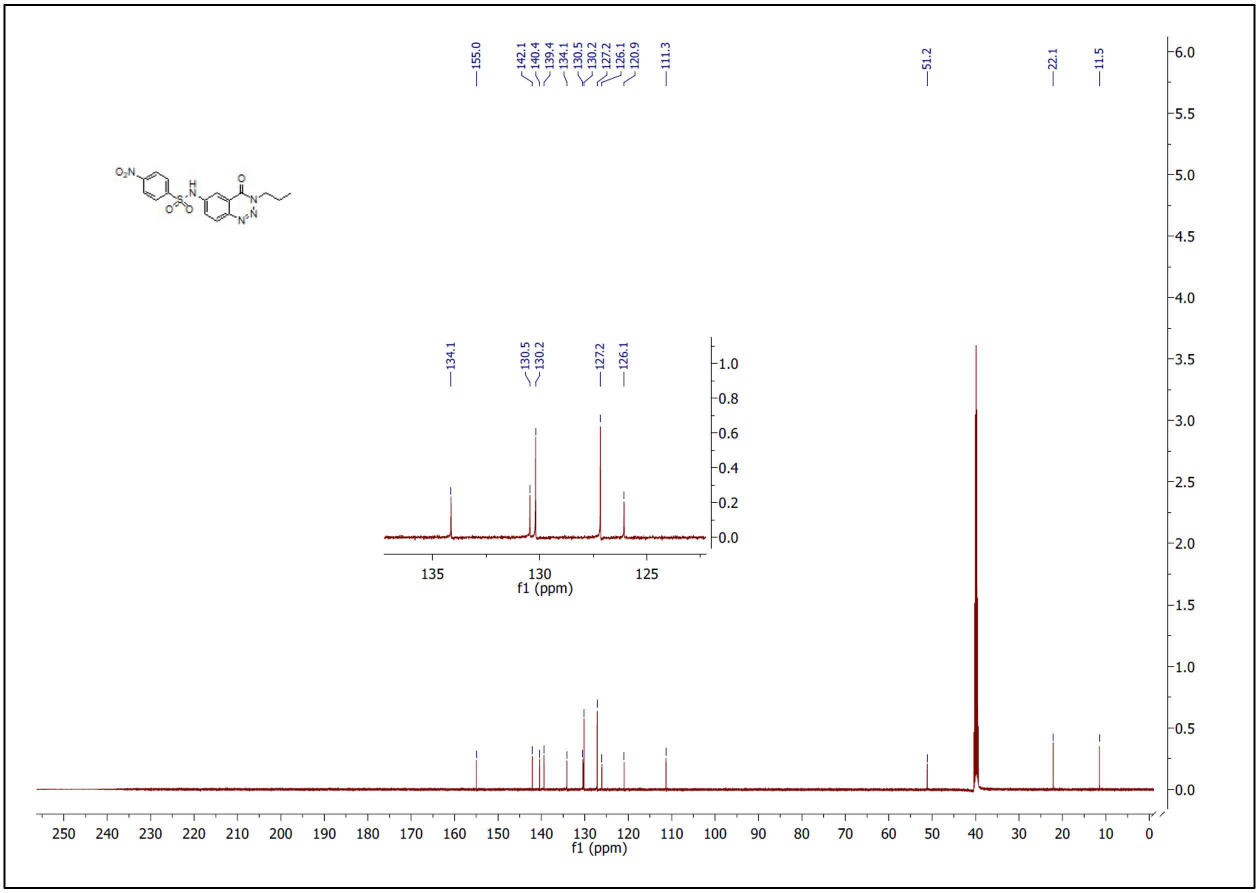

Figure S27. ^{13}C-NMR spectrum of **12e**.

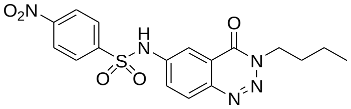

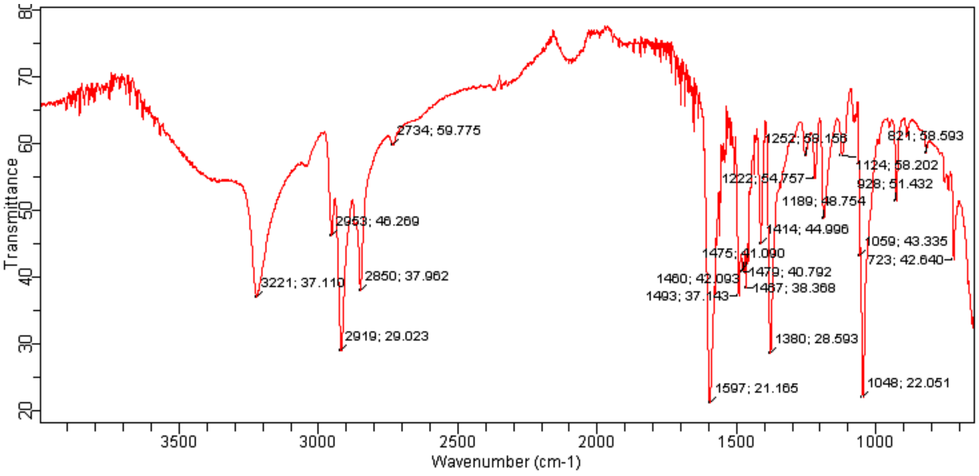

Figure S28. FTIR spectrum of **12f**.

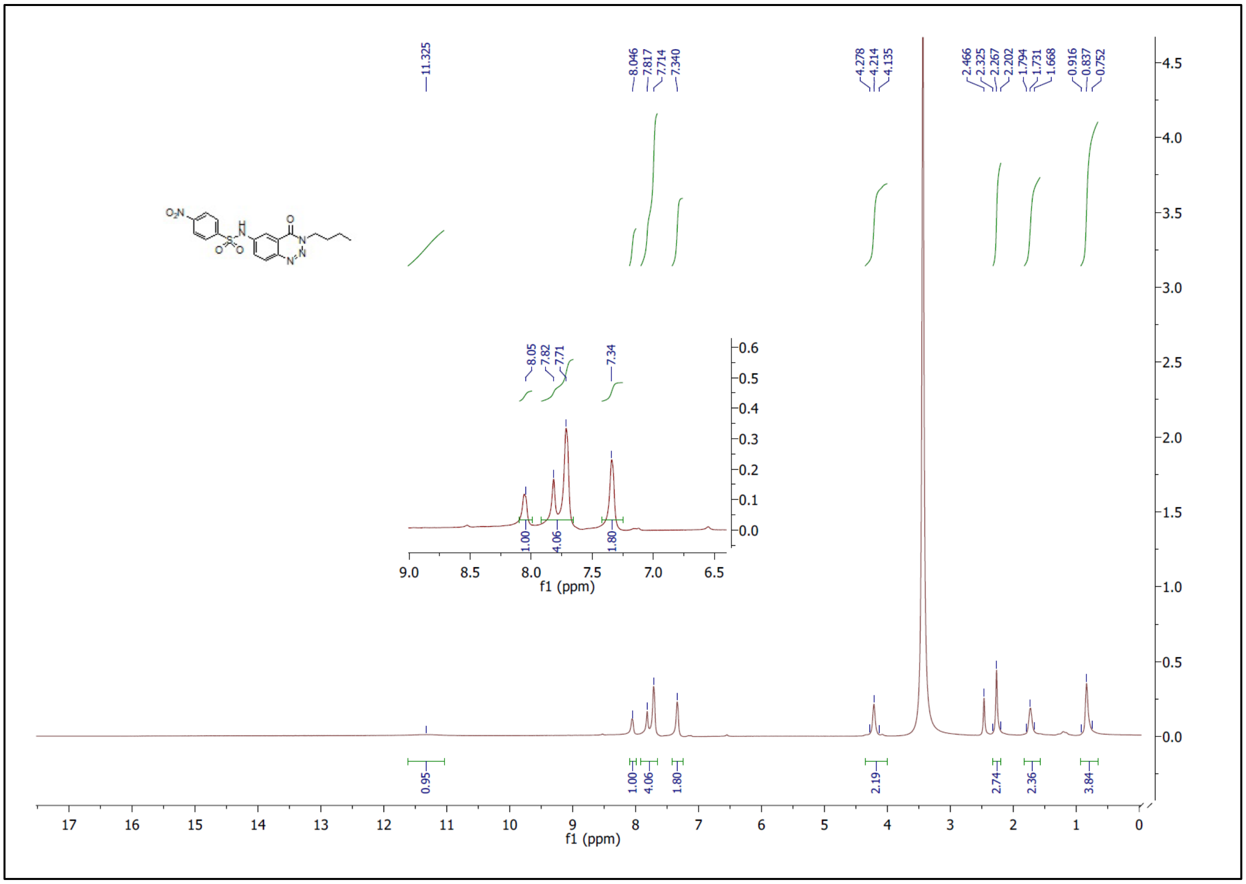

Figure S29. ^{1}H-NMR spectrum of **12f**.

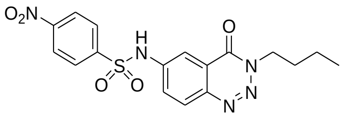

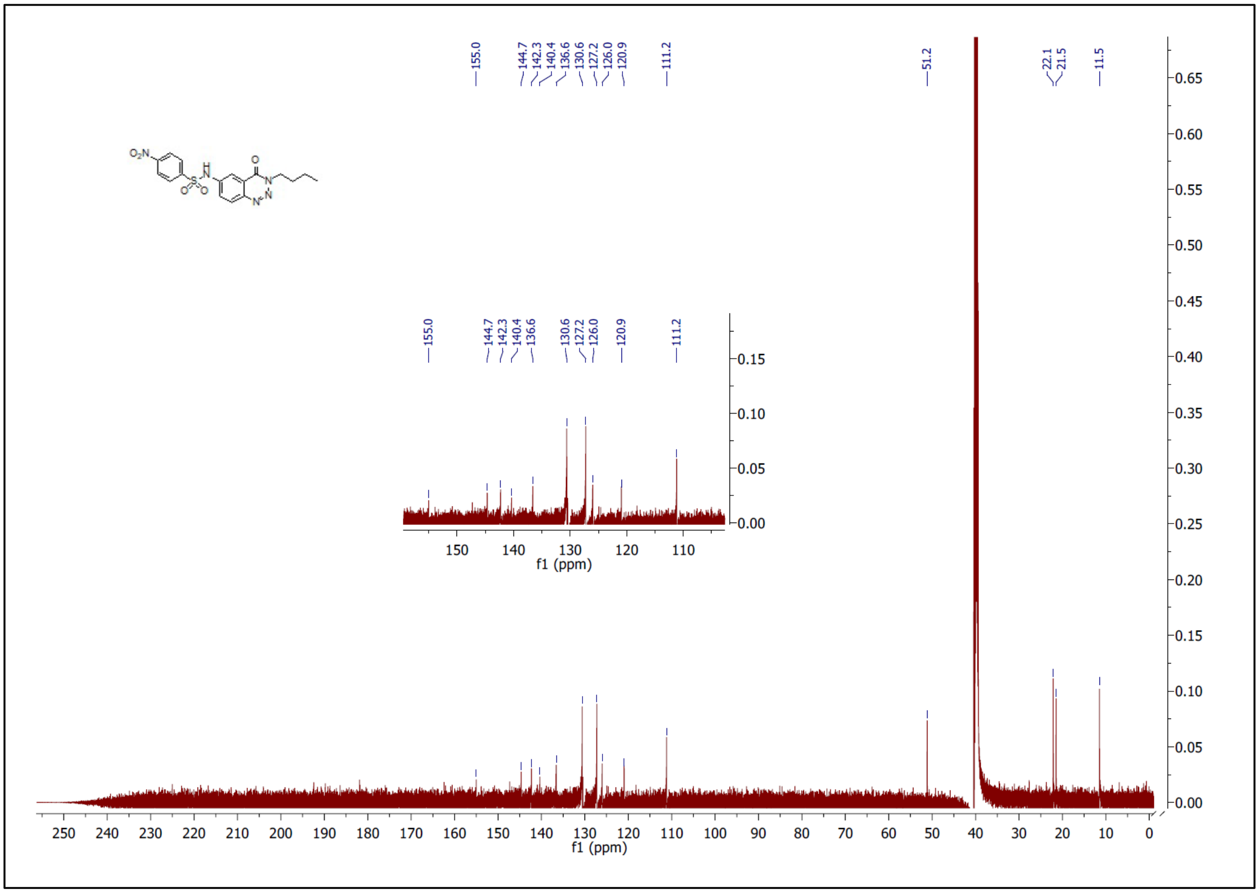

Figure S30. ^{13}C-NMR spectrum of **12f**.

2. Methodology for DFT Studies

The Gaussian 09 program has been used for the computational quantum analysis and calculations with electron correlation were carried out on the most reliable, Becke3-Lee-Yang-Parr (B3LYP) method along with 6-311G* basis set. The Gauss view software-generated pictures of all computed structures with their frontier orbital and molecular electrostatic potential (MEP) analysis. In the DFT method, HOMO and LUMO (highest occupied orbital and lowest unoccupied molecular orbital) are termed frontier molecular orbital (FMOs). According to Koopsman's theorem, ionization potential (I) and electron affinities (A) were calculated in an approximate negative value of HOMO energy (−*E* Homo) and negative value of the lowest unoccupied molecular orbital energy (−*E* Lumo). Some other electronic parameters of reactivity and compound interactions have been studied by chemical hardness ($\eta = E_{LUMO} - E_{HOMO}/2$), chemical softness ($S = 1/2\eta$), chemical potential [$\mu = -(E_{HOMO} + E_{LUMO})/2$], electronegativity ($\chi = E_{HOMO} + E_{LUMO}/2$) and electrophilicity [$\omega = (E_{HOMO} + E_{LUMO}/2)^2/2\eta$]. Variations in thermodynamic parameters and MEP (molecular electrostatic potential) maps have been evaluated by applying the same DFT method.

3. Binding energies of Docked Compounds

Table S1. Binding energies of the docked compounds 7a-f inside the active sites of *α*-glucosidase

Sr. No.	Compound	Compound free binding energy (kcal.mol^{-1})
1.	**12a**	−8.3
2.	**12b**	−8.7
3.	**12c**	−8.8
4.	**12d**	−8.8
5.	**12e**	−9.5
6.	**12f**	−9.3
